# Controlled-Radical Polymerization of α-Lipoic Acid: A General Route to Degradable Vinyl Copolymers

**DOI:** 10.1021/jacs.3c08248

**Published:** 2023-10-09

**Authors:** Kaitlin R. Albanese, Parker T. Morris, Javier Read de Alaniz, Christopher M. Bates, Craig J. Hawker

**Affiliations:** ^†^Department of Chemistry & Biochemistry, ^‡^Materials Research Laboratory, ^§^Materials Department, and ^∥^Department of Chemical Engineering, University of California, Santa Barbara, Santa Barbara, California 93106, United States

## Abstract

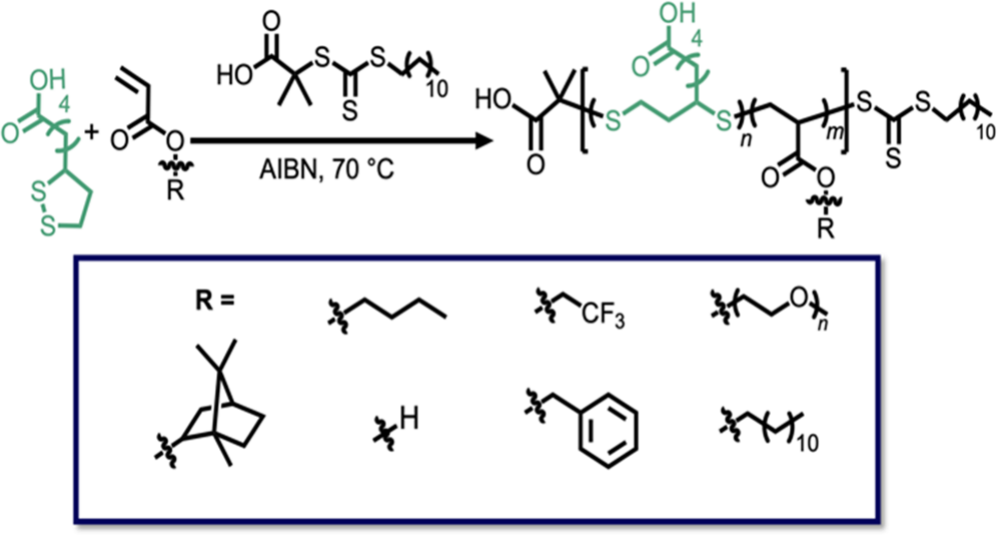

Here, we present the synthesis and characterization of statistical and block copolymers containing α-lipoic acid (LA) using reversible addition–fragmentation chain-transfer (RAFT) polymerization. LA, a readily available nutritional supplement, undergoes efficient radical ring-opening copolymerization with vinyl monomers in a controlled manner with predictable molecular weights and low molar-mass dispersities. Because lipoic acid diads present in the resulting copolymers include disulfide bonds, these materials efficiently and rapidly degrade when exposed to mild reducing agents such as tris(2-carboxyethyl)phosphine (*M*_n_ = 56 → 3.6 kg mol^–1^). This scalable and versatile polymerization method affords a facile way to synthesize degradable polymers with controlled architectures, molecular weights, and molar-mass dispersities from α-lipoic acid, a commercially available and renewable monomer.

## Introduction

The concept of controlled-radical polymerization has transformed polymer science, allowing for the synthesis of polymers with well-defined architectures, functional chain ends, predictable molecular weights, and narrow dispersities.^1−4^ The ability to control these parameters is crucial when designing polymers for a host of applications ranging from biomedicine to lithography and functional nanomaterials.^5−7^ Although the development of controlled-radical (co)polymerization has transformed polymer science and beyond, a major limitation of this technique relates to the use of traditional vinyl monomers that form backbones composed entirely of carbon–carbon bonds. As a result, vinyl polymers are difficult to degrade and create issues associated with long-lived plastic and rubber waste in the environment.

**Figure 1:**
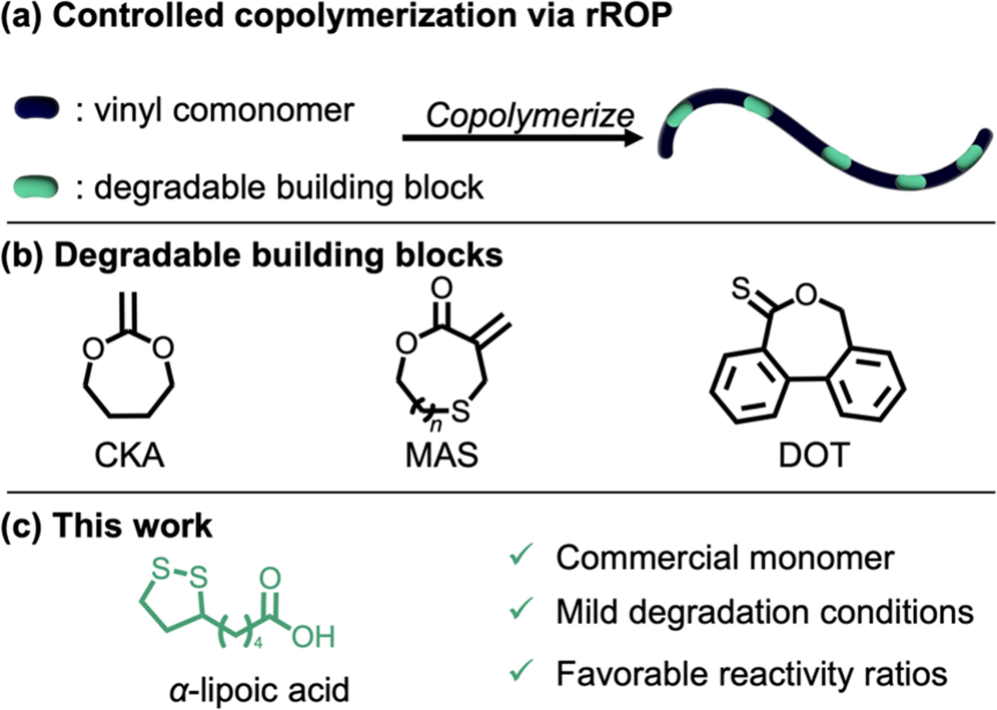
(a) Schematic of controlled copolymerization via rROP. (b) Building blocks previously used in controlled copolymerization impart degradable functionality. (c) This work uses α-lipoic acid as a commercially available, degradable building block in the controlled copolymerization of acrylate derivatives.

One solution to improve the sustainability of soft materials involves synthesizing degradable polymers containing cleavable functional groups along the polymer backbone, with polyesters^8,9^ and associated derivatives being prime examples.^10,11^ For common vinyl monomers such as acrylates that are broadly available and applicable, the lack of intrinsic degradability has necessitated the development of novel comonomers that undergo radical ring-opening polymerization (rROP) to impart degradability. Since the 1980s, this concept has gained significant attention as a method to incorporate degradable building blocks into the backbone of otherwise intractable vinyl-based polymers.^12,13^ A variety of cyclic compounds such as macrocyclic allyl sulfides (MAS)^14−18^ have emerged as candidates to impart degradability into various polymers. Another classic example is the copolymerization of acrylates with cyclic ketene acetals (CKA) as a method of introducing cleavable ester bonds.^19−25^ Despite their utility, CKAs have modest copolymerization reactivity leading to low yields and nonrandom incorporation. Recently, Gutekunst^26^ and Roth^27,28^ pioneered a thionolactone monomer, dibenzo[*c*,*e*]-oxepane-5-thione (DOT), that efficiently copolymerizes with acrylate derivatives and inserts degradable thioesters into the polymer backbone. Following these studies, the Guillaneuf^29^ and Johnson^30^ groups further extended the applicability of DOT by copolymerization with other monomer families such as styrenics. While pioneering, a challenge with all the systems described above is the necessity for multistep synthesis to prepare the cyclic comonomers (Figure 1).

Inspired by the aforementioned literature and a desire to simplify the synthesis of degradable comonomers for controlled rROP, we were drawn to α-lipoic acid (LA) – a commercially available and naturally occurring small molecule building block that is available on a multikilogram scale due to its wide use as a dietary supplement.^31−40^ LA features a 1,2-dithiolane ring that undergoes ring-opening polymerization in the presence of acid, base, or radicals, resulting in dynamic and degradable disulfide bonds.^41^ Tsarevsky^36,37^ and Endo^42,43^ previously demonstrated the rROP of LA derivatives with acrylates via a conventional free-radical process. Qu,^44^ Matile,^45,46^ Moore,^40^ and Waymouth^39^ extended the utility of LA through anionic and cationic ring-opening polymerization techniques.^47,48^

To demonstrate the utility of LA in designing functional, degradable materials, we present the controlled copolymerization of LA with a range of acrylate comonomers via reversible addition–fragmentation chain-transfer polymerization (RAFT). The resulting materials exhibit low molar-mass dispersities as well as excellent chain-end fidelity that enables reinitiation and block copolymer formation. Copolymerization kinetics were investigated to maximize the formation of degradable disulfide bonds along the polymer backbone. Upon exposure to mild reducing agents, these disulfide bonds are readily degraded, leading to oligomers with significantly reduced molar mass (e.g., *M*_n_ = 56 → 3.6 kg mol^–1^). In summary, the controlled-radical polymerization of α-lipoic acid creates opportunities to design and synthesize degradable polymers with a high degree of control over the architecture, molecular weight, and molar-mass dispersity using commercially available starting materials.

## Results and Discussion

**Figure 2:**
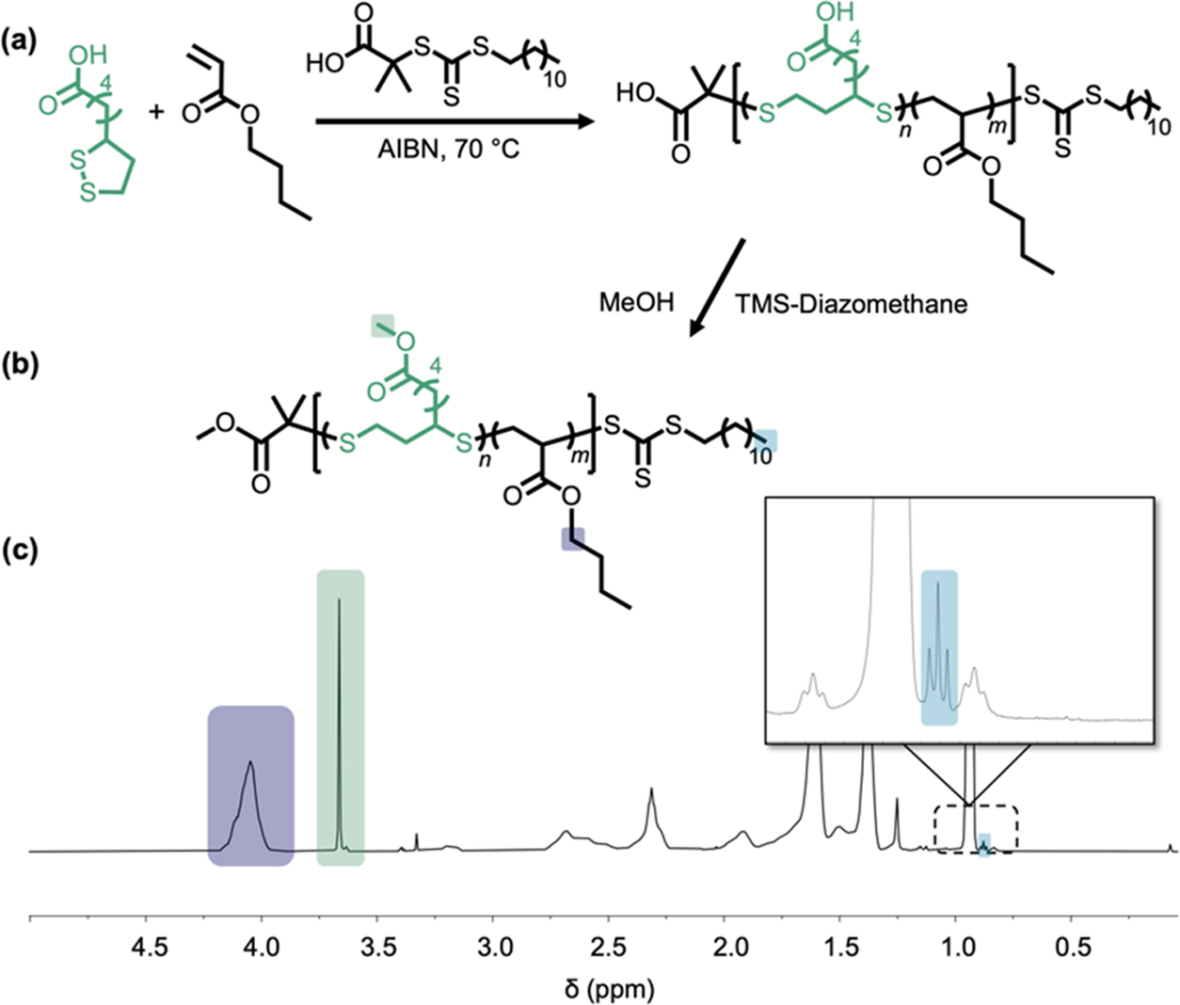
(a) Synthesis of *n*BA-*co*-LA. (b) Methylation of the *n*BA-*co*-LA copolymer for quantitative characterization of LA content. (c) ^1^H NMR (600 MHz) with unique resonances highlighted. Note that the rROP of LA is not regioregular.

We hypothesized that the controlled-radical polymerization of dl-α-lipoic acid (LA) would proceed most readily using RAFT in contrast to techniques involving metal catalysts such as atom-transfer radical polymerization (ATRP) that may suffer from sulfur chelation leading to uncontrolled or no polymerization.^49^ Initially, butyl acrylate (*n*BA) was selected as a model comonomer because of its ability to undergo copolymerization with LA via conventional (uncontrolled) conditions.^36,37,42^ 2-(Dodecylthiocarbonothioylthio)-2-methylpropionic acid (DTT), a trithiocarbonate chain transfer agent (CTA), was chosen for these copolymerizations of LA and *n*BA (Figures S1 and S2)^50^ as examined at 70 °C in the presence of azobis(isobutyronitrile) (AIBN, Figure 2a). To aid characterization, lipoic acid repeat units were quantitatively methylated by treatment with trimethylsilyldiazomethane to give the corresponding methyl esters. Following purification, the lipoic acid copolymer was analyzed via ^1^H NMR spectroscopy. Distinct resonances for both the lipoate (3.6 ppm) and butyl acrylate repeat units allowed for the LA content in these poly(butyl acrylate)-*co*-(α-lipoic acid) copolymers to be quantified (Figure 2b). In addition, ^1^H NMR spectroscopy indicates that the trithiocarbonate chain end remains intact (0.86 ppm, Figure 2c) with a broadening of the thioether resonances around 2.75 ppm resulting from ring-opening of the LA dithiolane ring. Size-exclusion chromatography (SEC) of the lipoate copolymers all show a monomodal distribution with low molar-mass dispersity (*Đ* = 1.08, Figure S2), further supporting a controlled polymerization. Notably, dithiobenzoate CTAs also lead to well-controlled copolymers (*M*_n_ = 8 kg mol^–1^, *Đ* = 1.11, Figure S3), whereas the use of dithiocarbamate and xanthate RAFT agents results in uncontrolled polymerization with high dispersities (Figures S4 and S5). Because RAFT is a controlled polymerization process, a range of molar masses (*M*_n_ = 12–56 kg mol^–1^) could be accurately targeted by varying the monomer-to-chain transfer agent, yielding well-defined copolymers with up to 30% LA in the feed (Figures S6 and S7 and Table S1). Further increasing the feed ratio of LA to 40 mol % was also successful, although lower monomer conversions were observed (Figure S8).

**Figure 3:**
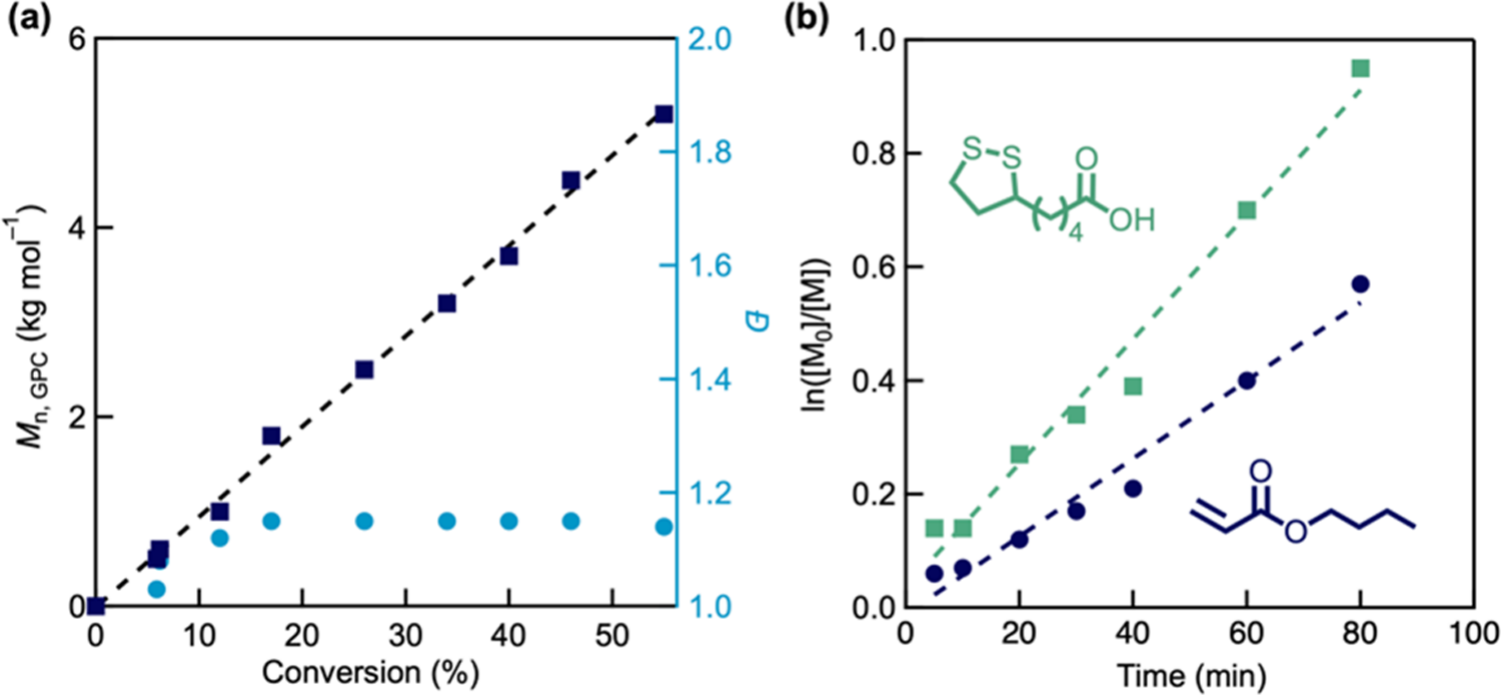
(a) Linear increase in overall molar mass as a function of conversion for the copolymerization of butyl acrylate and α-lipoic acid is consistent with a controlled polymerization. (b) First-order kinetics of *n*BA-LA copolymerization demonstrate LA incorporates faster than *n*BA, which favors the formation of disulfides along the backbone.

Livingness was further probed by using *n*BA (90%) and LA (10%) as a model system. A linear relationship was observed between the molar mass of the copolymer and conversion, resulting in first-order kinetics that are typical of controlled polymerizations (Figure 3a). A semilogarithmic plot derived from these kinetic experiments was used to monitor the incorporation of *n*BA and LA as the reaction proceeds (Figure 3b). The results indicate faster incorporation of lipoic acid compared to *n*BA with values similar to uncontrolled free-radical copolymerization of *n*BA and LA (Figure S9).^51^ This kinetic behavior not only favors LA incorporation but also is necessary for efficiently forming disulfides within diads along the polymer backbone that enable reductive degradation.

**Figure 4:**
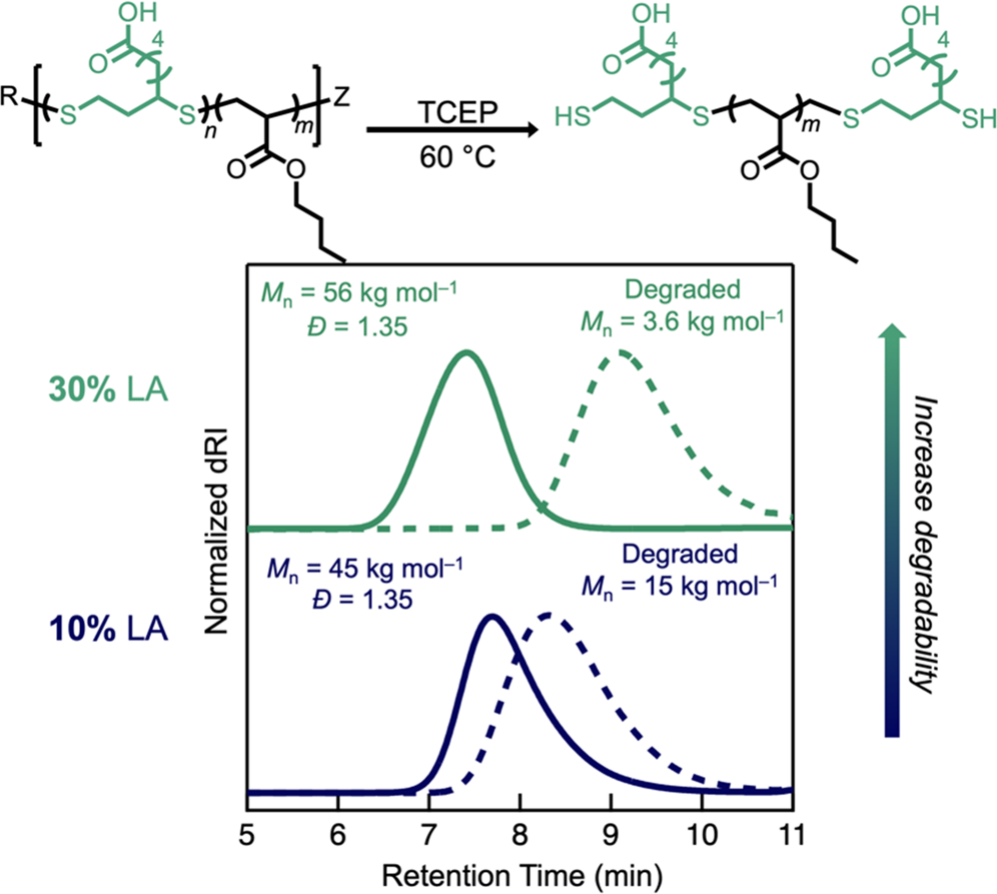
Degradability of LA-containing copolymers increases with the LA content in the feed.

To verify the presence of disulfide bonds along the backbone, poly(butyl acrylate*-co-*α-lipoic acid) copolymers were degraded with tris(2-carboxyethyl)phosphine (TCEP, 1 equiv relative to disulfide content), a mild reducing agent commonly used in biochemical applications.^52,53^ In a solution of THF/water (4:1) at 60 °C, the copolymer readily degrades into oligomers as evidenced by SEC (Figure 4 and Table 1). Alternative reagents are also capable of degrading disulfide bonds (e.g., NaBH_4_, Figure S14) and even the thioether bonds along the backbone are susceptible to degradation using AgNO_3_ (Figure S15).^54^

**Table 1:** Molecular Characterization of *n*BA-*co*-LA^a^

LA feed (%)	*M*_n,total_^b^	mol %_*n*Ba_^c^	mol %_LA_^c^	*Đ*^b^	*M*_n,deg_^b^
5	47	91	9	1.27	23
10	45	87	13	1.35	15
20	55	78	22	1.56	9.1
30	56	64	36	1.35	3.6

a Compositions are based on reactions stopped at 70% conversion.

b THF SEC analysis with PS standards and reported in kg mol^–1^.

c Determined using end-group analysis via ^1^H NMR and reported in kg mol^–1^.

Similar to previously reported studies, the degraded species can be repolymerized through oxidation of the reactive thiol chain ends.^30,51^ First, a discrete poly(butyl acrylate-*co*-methyl lipoate) (*n*BA-*co*-MLp) copolymer (*M*_n_ = 55 kg mol^–1^, *Đ* = 1.46) (see Supporting Information for further discussion, Figures S12 and S13) was synthesized via RAFT that on degradation with TCEP results in oligomers with decreasing molar mass (*M*_n_ = 11 kg mol^–1^, *Đ* = 1.70). After purification, the oligomers were oxidatively repolymerized using I_2_ and pyridine to reform disulfide bonds in a step-growth fashion to recover a high molar mass polymer (*M*_n_ = 66 kg mol^–1^, *Đ* = 1.78).

To further demonstrate the tunability of this system, *n*BA-*co*-LA with different feed ratios of LA was synthesized and degraded for analysis. As shown in Figure 4, increasing LA in the feed to 30 mol % proportionally increases the number of disulfides along the backbone (36 mol %) and yields even lower-molar-mass oligomers by SEC (*M*_n_ = 56 → 3.6 kg mol^–1^). This tunability provides control over the incorporation level of lipoic acid repeat units for targeted applications.

**Figure 5:**
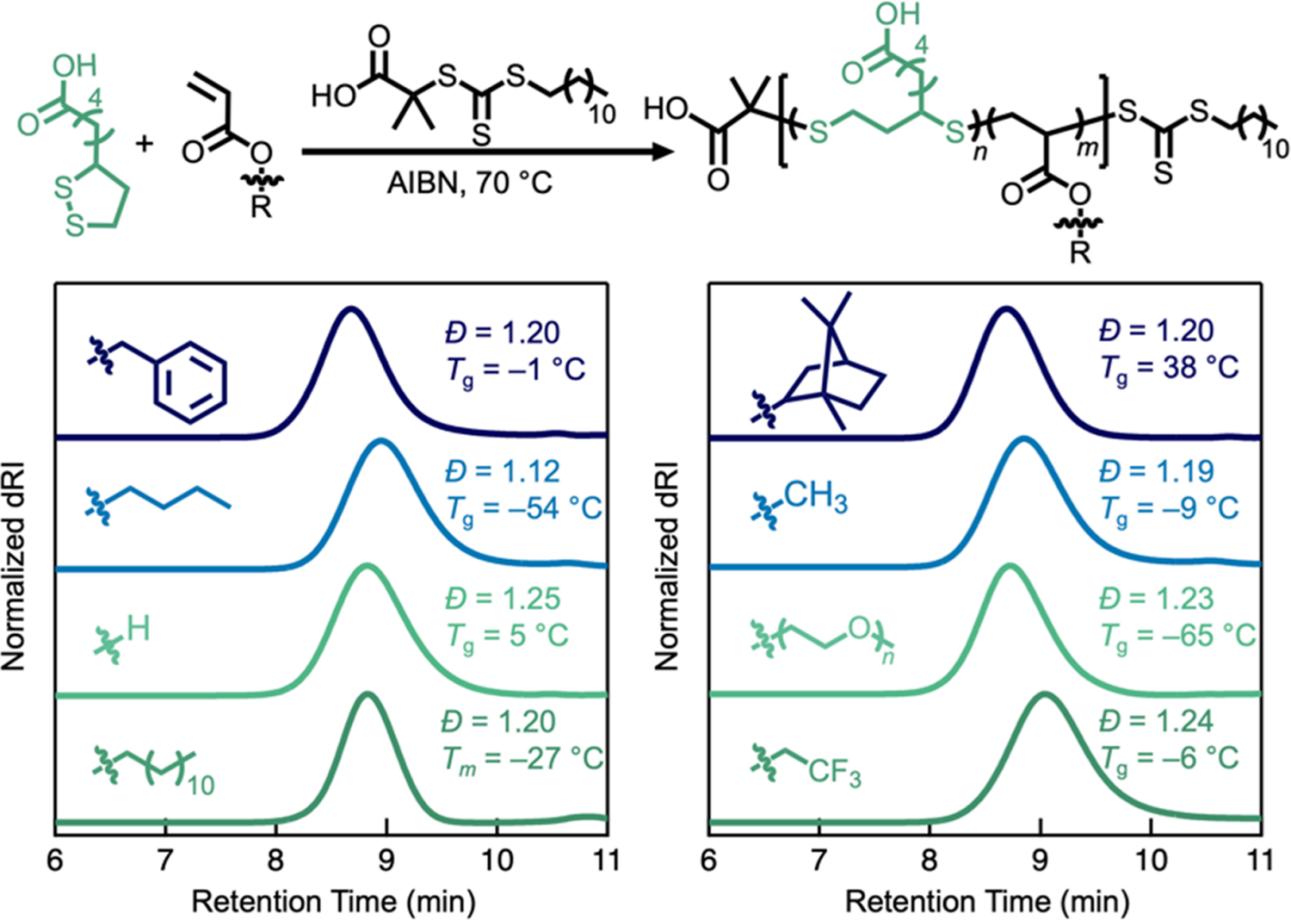
Efficient copolymerization of LA with a variety of acrylate comonomers. The acrylic acid copolymer was methylated prior to SEC analysis.

LA also smoothly and controllably copolymerizes with a variety of functional acrylate and acrylamide derivatives (Figure S17) to yield materials with a range of physical and chemical properties (Figure S18). For example, using a feed ratio of 30% LA, copolymers were prepared exhibiting low dispersity (*Đ* = 1.12–1.25) and efficient incorporation of LA (Figure 5 and Table 2). However, the radical reactivity of lipoic acid was not compatible with styrene and methyl methacrylate and resulted in homopolymerization of the respective vinyl monomers (Figures S20–S23).

**Table 2:** Molecular Characterization of the α-Lipoic Acid*-co*-Acrylate Copolymers^a^

acrylate	LA feed (%)	*M*_n,total_^b^	mol %_acrylate_^c^	mol %_LA_^c^	*Đ*^b^	*M*_n,deg_^b^
trifluoroethyl acrylate	30	10	66	34	1.24	2.1
butyl acrylate	30	6.2	65	35	1.12	1.8
isobornyl acrylate	30	11	69	31	1.20	5.0
acrylic acid	30	7.0	d	d	1.25	3.2
dodecyl acrylate	30	16	73	27	1.20	6.2
PEG-acrylate	30	8.2	58	42	1.23	7.0
benzyl acrylate	30	10	73	27	1.20	5.6
methyl acrylate	30	9	78	22	1.19	4.6

a Compositions are based on reactions stopped at 70% conversion.

b THF SEC analysis with PS standards in kg mol^–1^.

c Determined using end-group analysis via ^1^H NMR and reported in kg mol^–1^.

d No unique resonance in the ^1^H NMR spectrum.

**Figure 6:**
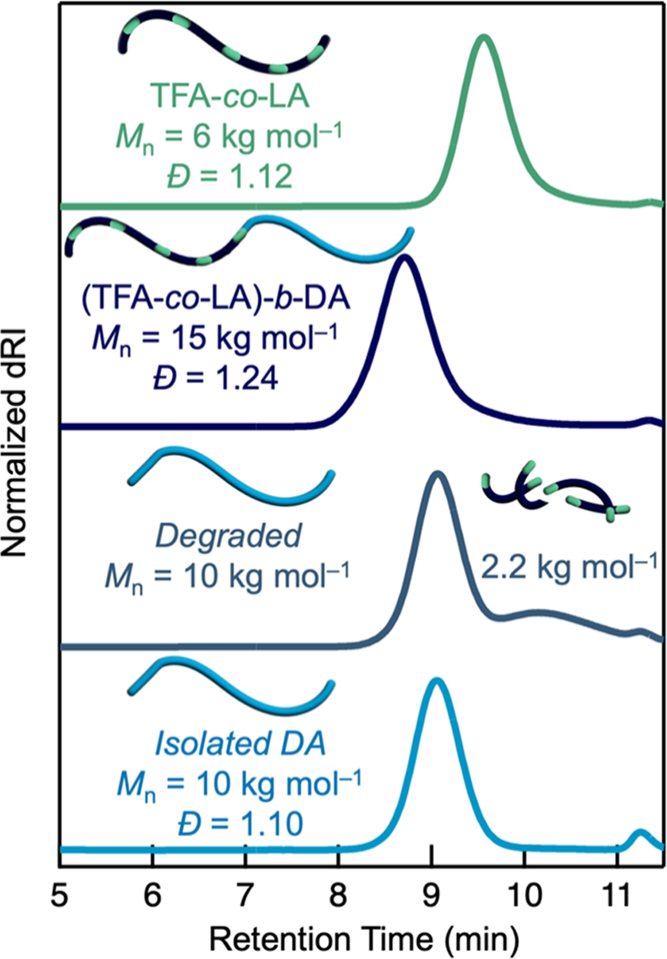
Controlled polymerization of LA enables the synthesis of degradable block copolymers. SEC traces of TFA-*co*-LA (top), the (TFA-*co*-LA)-*b*-DA diblock (middle), the degraded diblock copolymer (middle), and the isolated DA homopolymer (bottom).

To further accentuate the utility of using controlled polymerizations to create LA-based materials, we synthesized a variety of degradable block copolymers that would otherwise be inaccessible using conventional free-radical polymerization. First, chain extension of a poly(trifluoroethyl acrylate–*co*–α-lipoic acid) macroinitiator (TFA-*co*-LA, 6 kg mol^–1^, *Đ* = 1.12) with dodecyl acrylate (DA) yielded the corresponding diblock copolymer, poly[(trifluoroethyl acrylate–*co*–α-lipoic acid)–*block*–dodecyl acrylate] (Figure S24). As depicted in Figure 6, SEC analysis showed a clear shift to lower retention times and higher molar mass (*M*_n_ = 15 kg mol^–1^) upon chain extension when compared to the starting lipoic acid copolymer with little or no residual macroinitiator. This result indicates excellent end-group fidelity of the starting macroinitiator. Furthermore, when the diblock copolymer was treated with TCEP and analyzed by SEC, a bimodal distribution was observed. At higher retention times, a broad peak corresponding to the degraded lipoic acid copolymer was observed (*M*_n_ = 2.2 kg mol^–1^, *Đ* = 1.7) with the remaining dodecyl acrylate block appearing as a well-defined, low dispersity peak (*M*_n_ = 10 kg mol^–1^, *Đ* = 1.10). This behavior is expected, given the presence of only vinyl (C–C) repeat units in the dodecyl acrylate block. Significantly, after fractional precipitation in methanol, the resulting dodecyl acrylate block (*M*_n_ = 10 kg mol^–1^, *Đ* = 1.10) appears almost indistinguishable from its distribution in the crude reaction mixture (after degradation, Figure 6). The observation of characteristic resonances for dodecyl acrylate (4.0 ppm) and the absence of a unique resonance at 4.5 ppm for TFA repeat units via ^1^H NMR further supports the controlled incorporation of LA units in only one block during the RAFT block copolymerization process (Figure S25).

**Figure 7:**
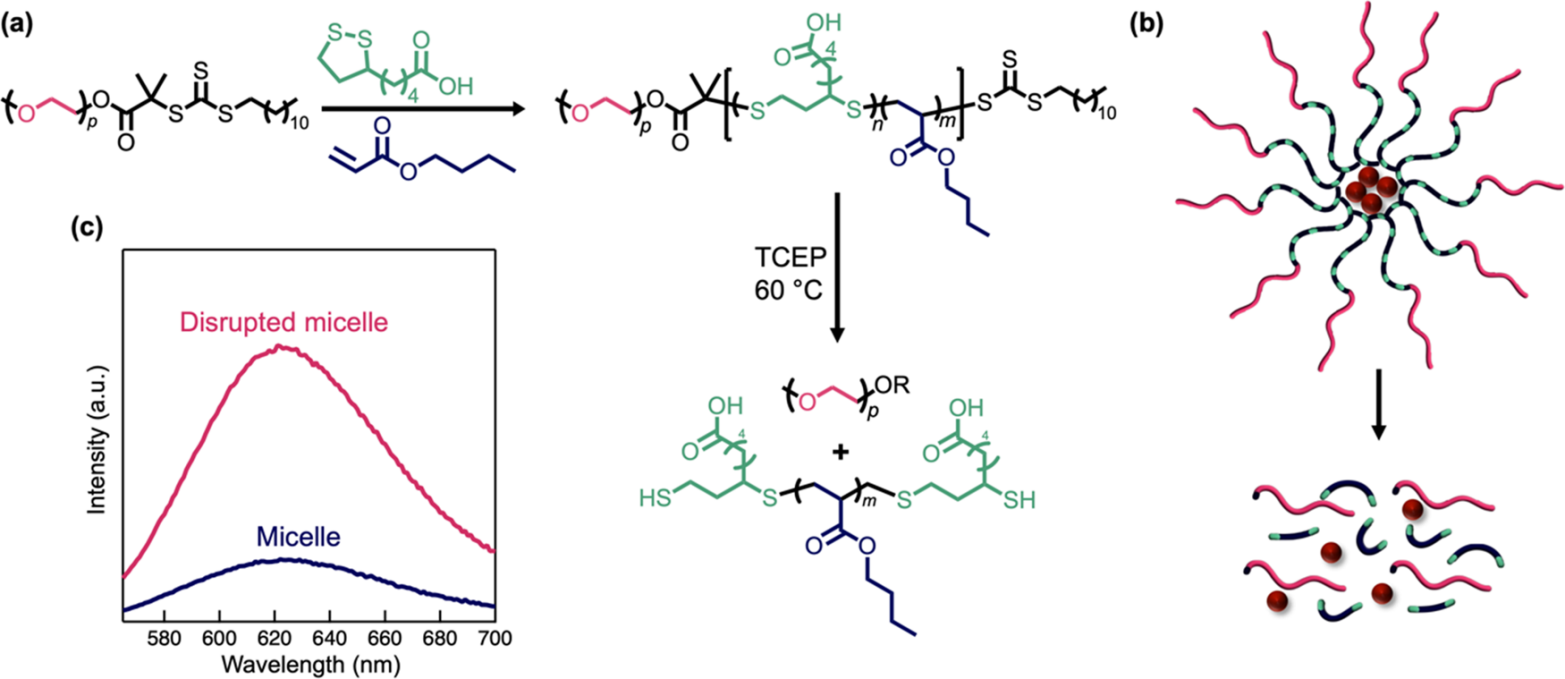
(a) Synthesis of micelle-forming diblock copolymers, PEG-*b*-(*n*BA-*co*-LA), that are degradable in aqueous solution. (b) Illustration of self-assembly in water to encapsulate Nile red, a hydrophobic dye (represented graphically as red spheres) that releases upon degradation of the core. (c) Photoluminescence of micelles before and after degradation.

To further illustrate the ability to prepare degradable systems, amphiphilic block copolymers were designed based on poly(ethylene glycol) (PEG). Upon self-assembly in aqueous solutions, micelles form with a PEG corona and a degradable, hydrophobic core that can encapsulate and then release a hydrophobic dye, Nile red. In a stepwise fashion, the RAFT chain-transfer agent DTT was coupled to monomethoxy PEG (*M*_n_ = 1.8 kg mol^–1^, *Đ* = 1.04) followed by chain extension with a mixture of *n*BA (80%) and LA (20%) to yield PEG-*b*-(*n*BA-*co*-LA) diblock copolymers (*M*_n_ = 8.4 kg mol^–1^, *Đ* = 1.08, see Figures 7a and S26–S30). In an aqueous medium containing Nile red, the diblock copolymer self-assembles into micelles (Figure S31), with the disassembly process monitored via the photoluminescence of Nile red before and after degradation (see Supporting Information). Before degradation, the micelle assembly shows a weak emission (λ = 624 nm) that is consistent with Nile red molecules confined to the hydrophobic interior of the micelle cores (Figure 7b,c). After degradation and cleavage of the LA-containing domains, the intensity increased dramatically (∼10-fold) as a result of Nile red molecules being released from the disrupted micelles.^55^

## Conclusions

In conclusion, we have demonstrated the controlled-radical ring-opening copolymerization of α-lipoic acid – a commercially available and degradable building block – with a variety of acrylate and acrylamide comonomers via RAFT. The resulting copolymers exhibit low dispersities (*Đ* = 1.1–1.3) and tunable molecular weights (*M*_n_ = 6–56 kg mol^–1^), with the reactivity of lipoic acid favoring the formation of degradable lipoic acid–lipoic acid diads along the backbone. As a result, these copolymers readily degrade into low–molecular-weight species under mild reducing conditions (e.g., *M*_n_ = 56 → 3.6 kg mol^–1^). The molar mass after degradation can be tuned by changing the feed ratio of LA (up to 30%) during the polymerization. In summary, the controlled-radical ring-opening of lipoic acid is compatible with a wide variety of vinyl comonomers and represents a scalable and versatile synthetic platform for controlling the degradability of polyacrylates – a popular family of materials across many applications. While compatible vinyl comonomers are currently limited to acrylates and acrylamides, this work can be used to design other ring-opening monomers based on a dithiolane motif that may undergo copolymerization with a wider range of vinyl monomer families.

## Supporting Information Available

The Supporting Information is available free of charge at https://pubs.acs.org/doi/10.1021/jacs.3c08248. All curated data are available in the manuscript or the Supporting Information. Raw data are available from a permanent online repository at DOI: 10.5061/dryad.dr7sqvb49.

Reagent information, size-exclusion chromatograms, and ^1^H NMRs of copolymers (Figures S1–S8, S10–S17, and S20–S30), kinetic plots (Figure S9), differential scanning calorimetry of materials (Figure S18), thermogravimetric analysis (Figure S19), and dynamic light scattering (Figure S31) (PDF)
