# Supplementary material for: Controlled-Radical Polymerization of α-Lipoic Acid: A General Route to Degradable Vinyl Copolymers
Source: J Am Chem Soc. 2023 Oct 9;145(41):22728–34. doi: 10.1021/jacs.3c08248 (PMC10591472; doi:10.1021/jacs.3c08248)
Supplement: Supplementary file 1 — ja3c08248_si_001.pdf [file ja3c08248_si_001.pdf]

**Controlled Radical Polymerization of *α*-Lipoic Acid: A General Route to Degradable Vinyl Copolymers**

Kaitlin R. Albanese,[+,†] Parker T. Morris,[+,†] Javier Read de Alaniz,*[+] Christopher M. Bates,*[,†,‡] and Craig J. Hawker*[,†,‡]

[+]*Department of Chemistry & Biochemistry,* [†]*Materials Research Laboratory,* [‡]*Materials Department, and* [§]*Department of Chemical Engineering, University of California, Santa Barbara, California 93106, United States*

## Materials

All reagents were used as received except where noted. DL-α-lipoic acid (LA), tris(2-carboxyethyl)phosphine hydrochloride (TCEP), 2,2,2-Trifluoroethyl acrylate (FA), isobornyl acrylate (IA), dodecyl acrylate (DA), benzyl acrylate (BA), polyethylene glycol acrylate (PA), poly(ethylene glycol) methyl ether (PEG), and butyl acrylate (*n*BA) were purchased from TCI chemicals. Tetrahydrofuran (THF), and (Trimethylsilyl)diazomethane (TMS-diazomethane) were purchased from Fisher Scientific. 2-(Dodecylthiocarbonothioylthio)-2-methylpropionic acid (DTT), 2-Cyano-2-propyl benzodithioate (CBT), Cyanomethyl methyl(phenyl)carbamodithioate (CMD), dimethyl acrylamide, styrene and acrylic acid (AA) were purchased from Sigma Aldrich. Deuterated solvents were acquired from Cambridge Isotope Laboratories. Vinyl monomers were passed through a column of basic alumina to remove the inhibitor prior to use. 2-((ethoxycarbonothioyl)thio)propanoic acid (ECT) was synthesized from a modified literature procedure.[1] MWCO 1 kDa dialysis tubing from Spectrum Laboratories was used for copolymer purification when stated.

## Molecular Characterization

### *$^1$H nuclear magnetic resonance spectroscopy*

Solution state $^1$H nuclear magnetic resonance (NMR) spectra were recorded on a Varian VNMRS 600 MHz spectrometer. Chemical shifts ($\delta$) are reported in ppm relative to residual protio solvent in $CDCl_3$ (7.26 ppm).

### *Size-exclusion chromatography instrumentation*

Size-exclusion chromatography (SEC) was performed on a Waters instrument using a differential refractive index detector and two Tosoh columns (TSKgel SuperHZM-N, 3 µm polymer, 150 × 4.6 mm) with THF at 35 °C or chloroform containing 0.25% TEA at 35 °C for the mobile phase. Molar masses and molar mass dispersities ($Đ$) were determined against narrow PS standards (Agilent).

*Dynamic Light Scattering (DLS)*

The DLS experiment was performed with disposable 4 mL plastic cuvettes for the DLS instrument with 1 mL of aqueous solution. The light scattering signals were measured by using a Marvin Instrument Ltd. nanoZS Zetasizer.

*Photoluminescence*

Solution-state photoluminescent data were obtained using a Jobin-Yvon HORIBA FluoroMax-4 (xenon source, 1.0 nm excitation, and emission slit widths, 1 nm step size, $\lambda_{excitation}$ = 536 nm) equipped with a solution-state sample holder and quartz cuvette with a diameter of 1 cm. Photoluminescent data were analyzed using the FluorEssence (v3.5) software powered by Origin. Samples were prepared according to the following: A stock solution of polymer (~2.2 mg) was dissolved in 100 µL of THF. The micelles were prepared by rapidly mixing the THF solution with 3 mL of Milli-Q water. With Nile red, a 1 µg/mL solution was added to the polymer THF solution prior to micelle formation.

*Thermal characterization*

Thermogravimetric analysis (TGA) was performed under air on a TA Instruments Q500 at a heating rate of 10 °C $min^{-1}$ with a sample size of ca. 4 mg. Differential Scanning Calorimetry (DSC) was performed using a TA Instruments DSC Q2000 at a heating/cooling rate of 10 °C/min using 3–5 mg of sample in a sealed aluminum pan.

*General synthesis of degradable copolymers (nBA-co-LA) 9:1*

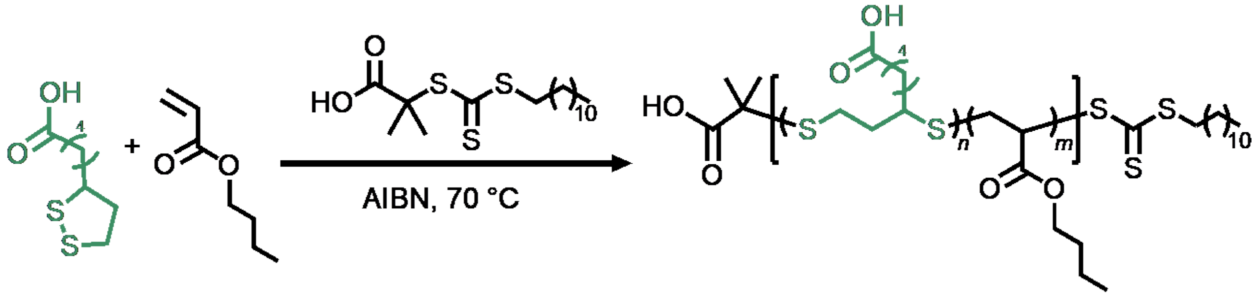


A stock solution of AIBN was used for all RAFT polymerizations (4.2 mg $mL^{-1}$).

DTT (0.020 g, 0.05 mmol) was added to a pressure vessel with LA (0.14 g, 0.69 mmol), *n*BA (0.80 g, 6.24 mmol), AIBN stock solution (97 µL, 0.003 mmol), and 0.85 mL of THF. The reaction mixture was purged with Argon gas for 15 min and then placed in a 70 °C oil bath. The conversion was monitored via $^1$H NMR and stopped at 50% – 70% monomer conversion. The reaction vessel was quenched on ice and purified by dialysis in acetone (1 L × 2). Precipitation was not the preferred method of purification due to the partial solubility of the lipoic acid in most solvents resulting in a drastic loss of product. Low conversions were targeted to preserve high chain-end fidelity.

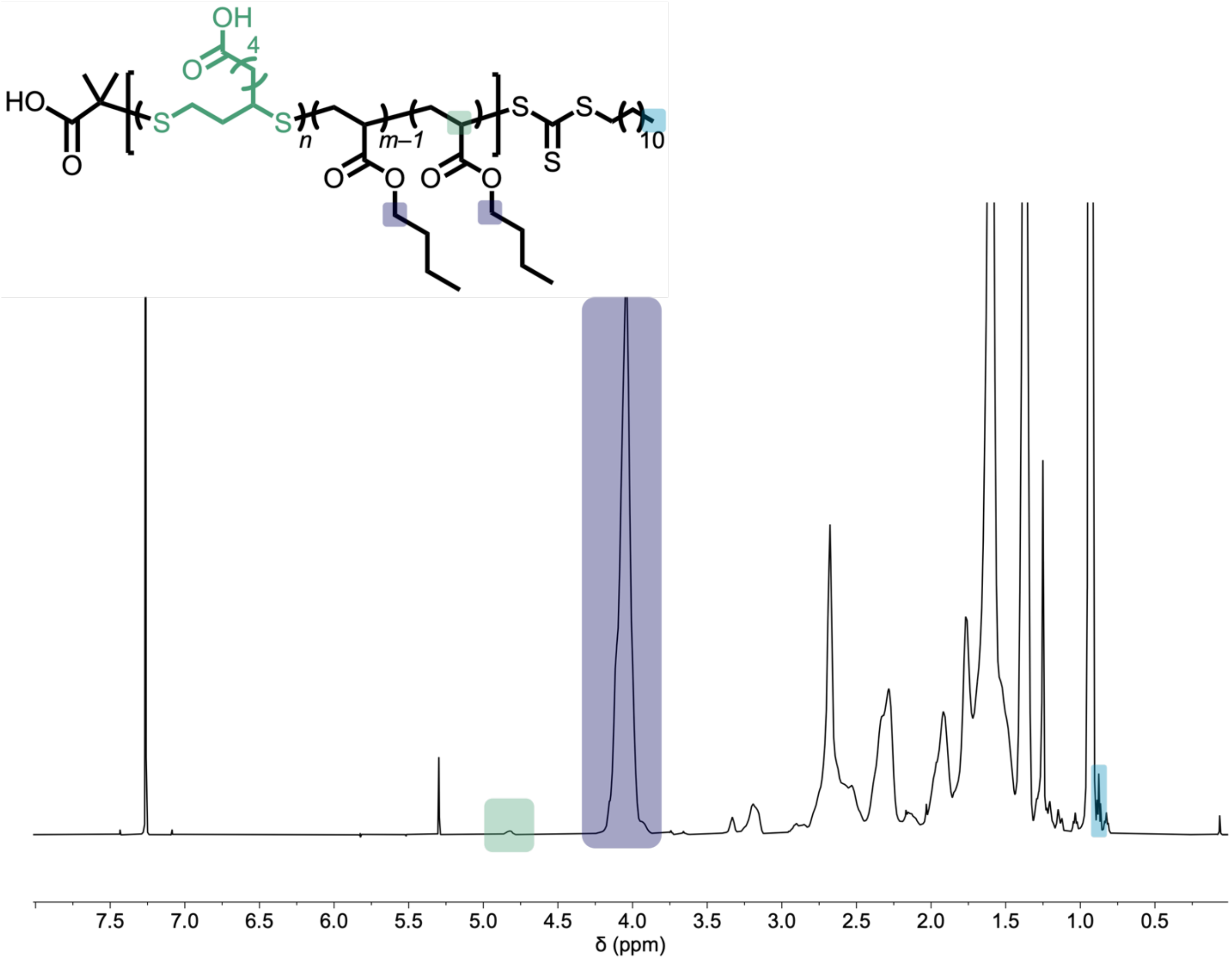


**Figure S1.** $^{1}$H NMR analysis of *n*BA-*co*-LA with characteristic resonances highlighted.

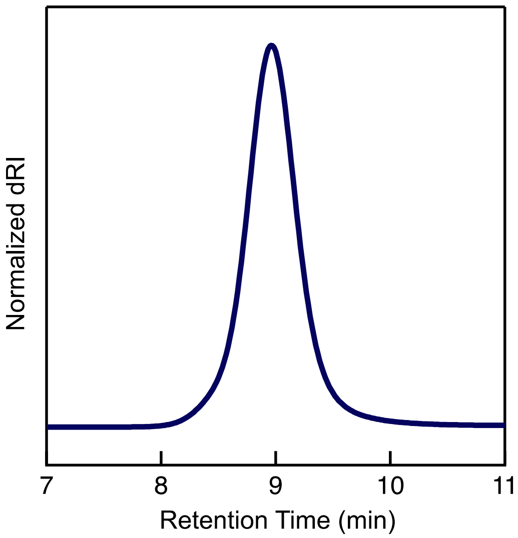


**Figure S2.** SEC trace with normalized differential refractive index (dRI) detection for *n*BA-*co*-LA. $M_n$: 13 kg mol$^{-1}$, $Đ$ = 1.08.

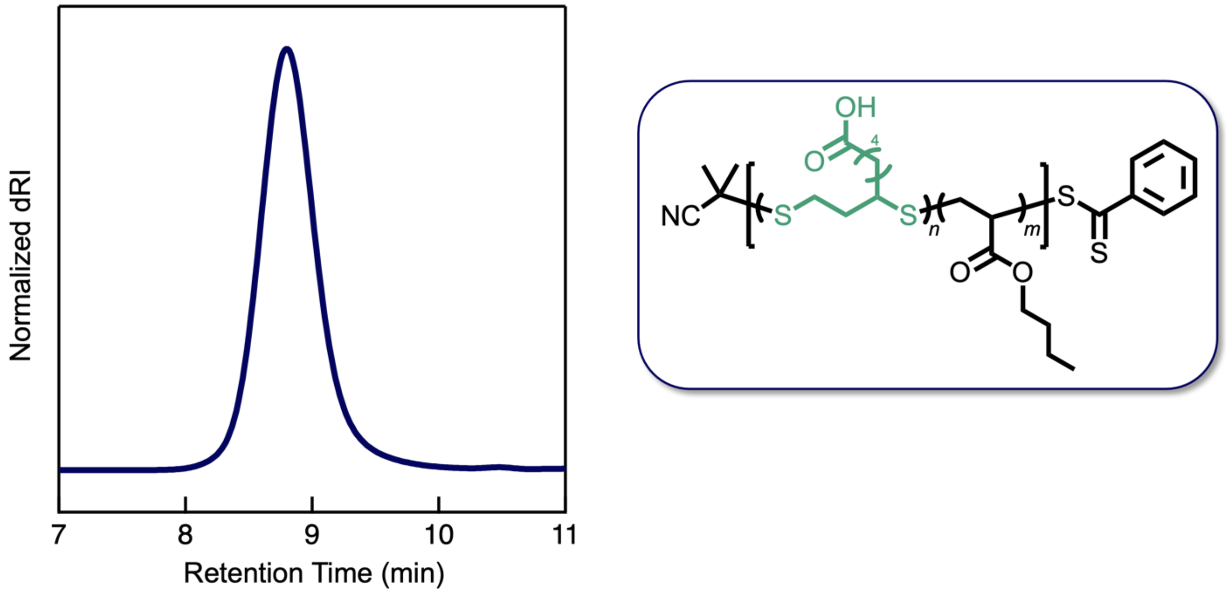


**Figure S3.** SEC trace with normalized differential refractive index (dRI) detection for *n*BA-*co*-LA synthesized with CBT. $M_n$: 8 kg mol$^{-1}$, $Đ$ = 1.11.

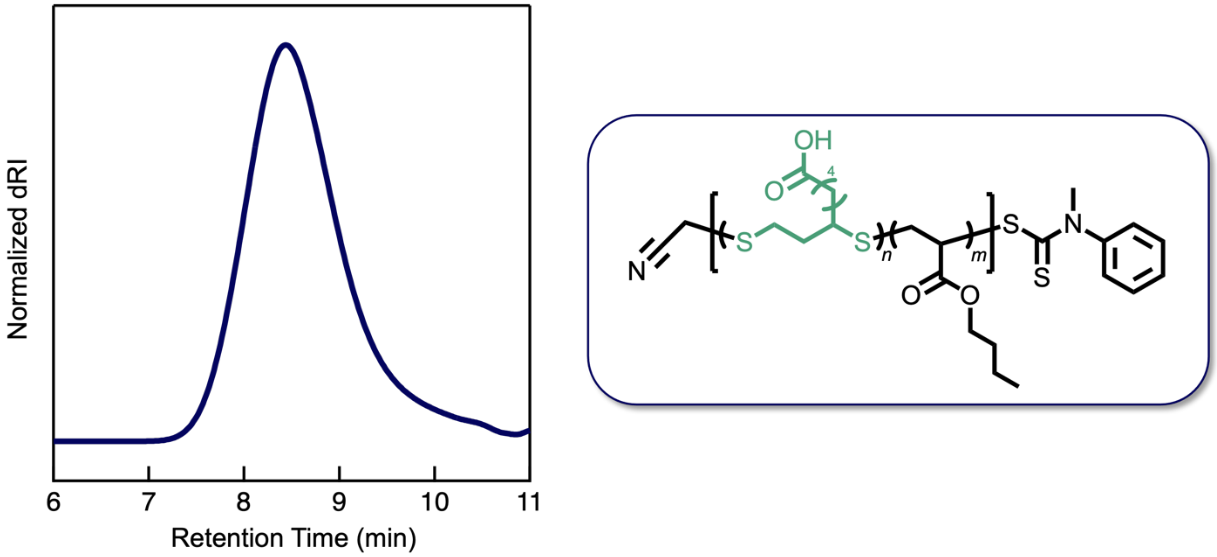


**Figure S4.** SEC trace with normalized differential refractive index (dRI) detection for *n*BA-*co*-LA synthesized with CMD. $M_n$: 11 kg mol$^{-1}$, $Đ$ = 1.47.

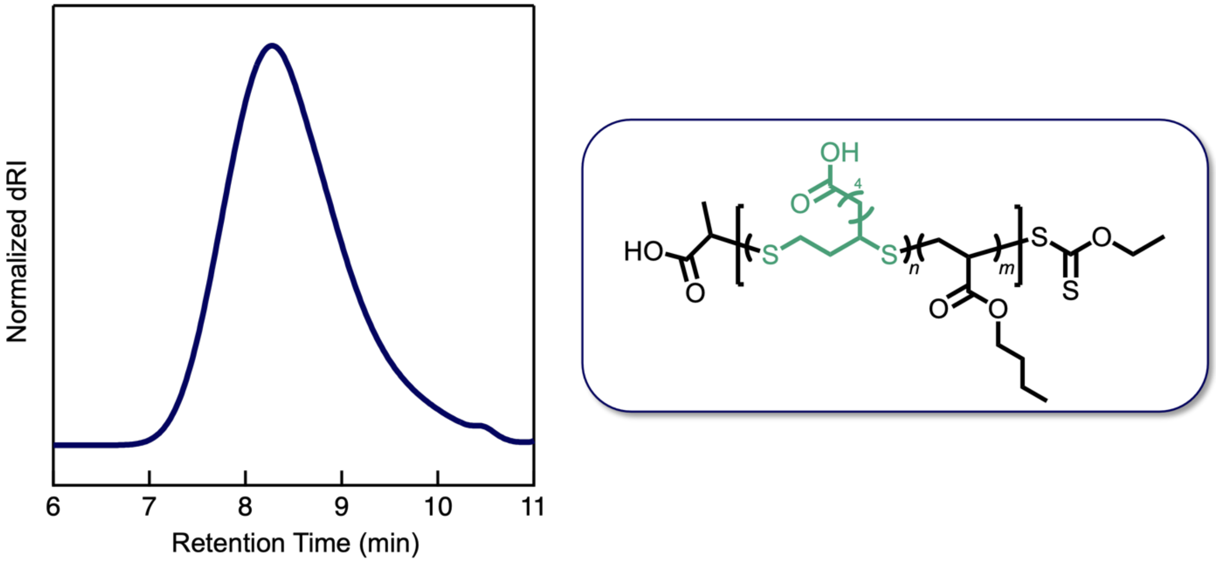


**Figure S5.** SEC trace with normalized differential refractive index (dRI) detection for *n*BA-*co*-LA synthesized with ECT. $M_n$: 13 kg mol$^{-1}$, $Đ$ = 1.5.

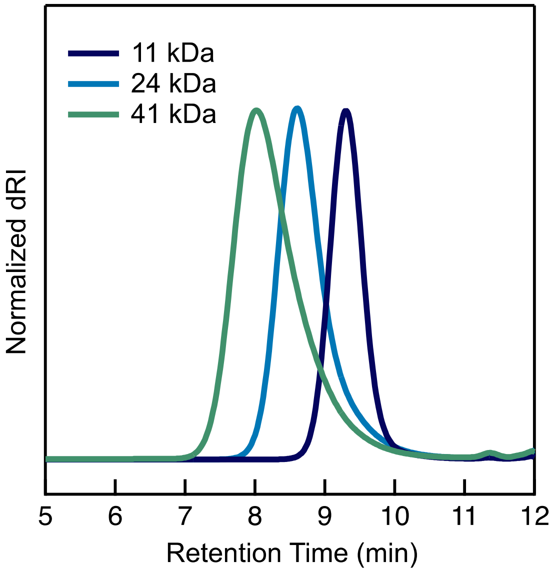


**Figure S6.** SEC trace with normalized differential refractive index (dRI) detection for *n*BA-*co*-LA with 10% feed of LA.

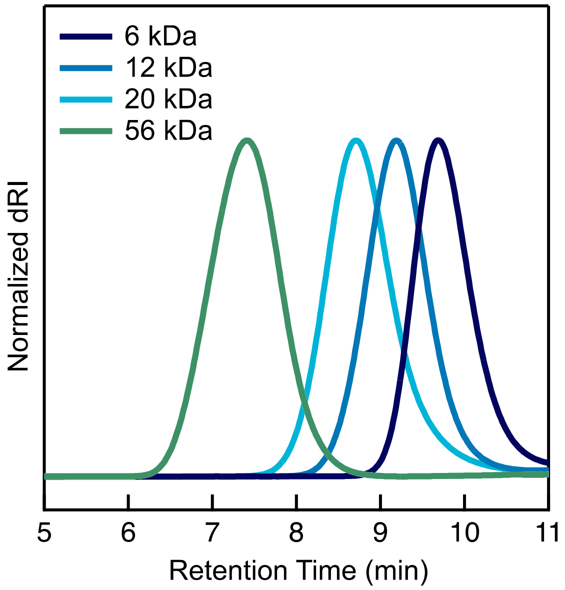


**Figure S7.** SEC trace with normalized differential refractive index (dRI) detection for *n*BA-*co*-LA with 30% feed of LA.

**Table S1.** Molecular characterization of *n*BA-*co*-LA.

| Entry | LA feed | $M_{n,total}^{a}$ | $M_{n,Deg}^{a}$ | $Đ^{b}$ | Target DP |
|---|---|---|---|---|---|
| 1 | 10% | 11 | 5.6 | 1.08 | 74 |
| 2 | 10% | 24 | 11 | 1.12 | 184 |
| 3 | 10% | 41 | 15 | 1.35 | 368 |
| 5 | 30% | 12 | 2.1 | 1.18 | 66 |
| 6 | 30% | 20 | 2.6 | 1.26 | 165 |
| 7 | 30% | 56 | 3.0 | 1.35 | 330 |

[a] THF SEC analysis with PS standards in kg $mol^{-1}$. [b]Determined using end-group analysis via $^{1}H$ NMR and reported in kg $mol^{-1}$. Compositions are based on reactions quenched at 70% conversion.

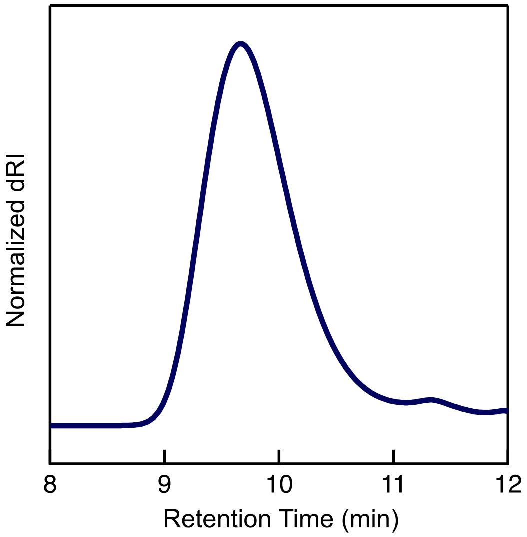


**Figure S8.** SEC trace with normalized differential refractive index (dRI) detection for *n*BA-*co*-LA with 40% feed of LA. $M_n$: 4.5 kg $mol^{-1}$, $Đ$ = 1.42.

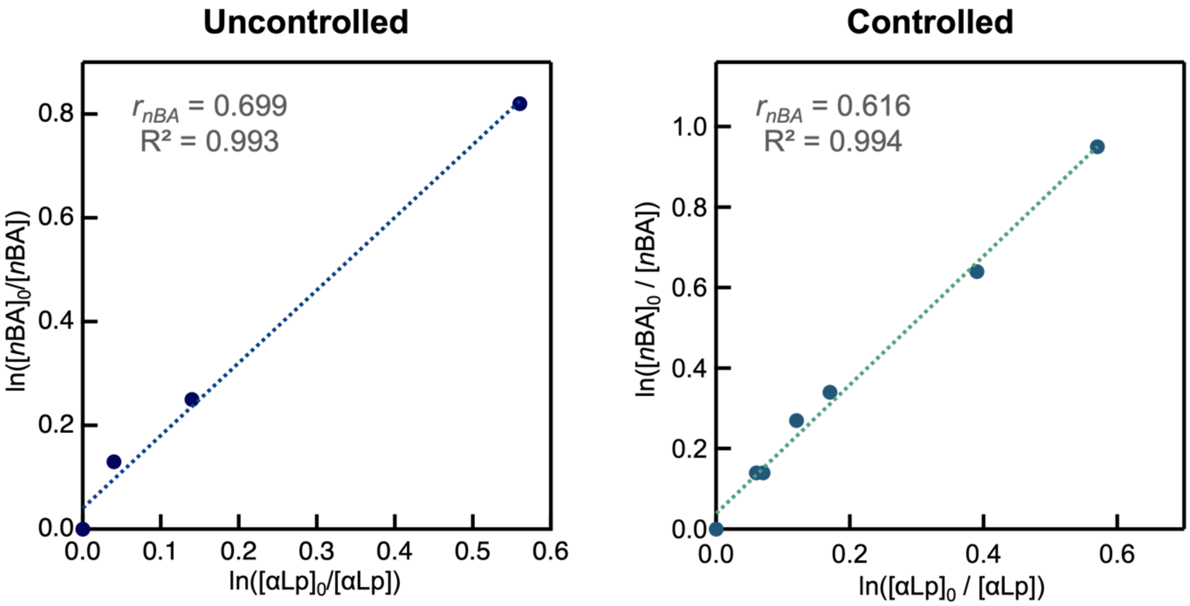


**Figure S9.** Uncontrolled vs. controlled reactivity ratios of *n*BA for the *n*BA-LA copolymerization.

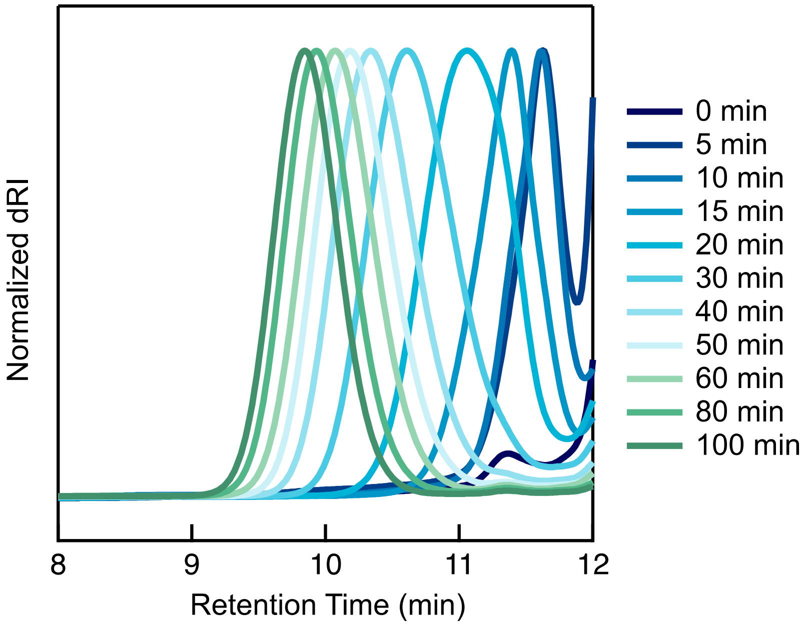


**Figure S10.** SEC trace with normalized differential refractive index (dRI) detection for *n*BA-*co*-LA copolymerization. Each trace depicts a time point taken during the copolymerization reaction.

Degradation *of nBA-co-LA with TCEP*

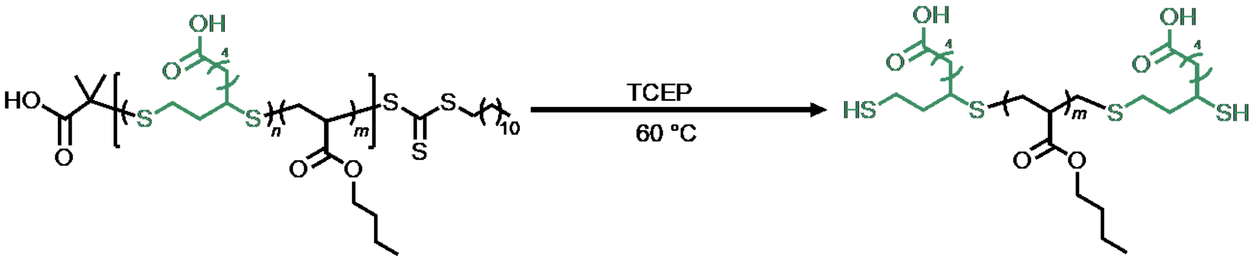


In a 4 mL dram vial, *n*BA-*co*-LA (150 mg, 0.09 mmol) was dissolved in minimal THF. A solution of TCEP (5 equiv. to thiol) in THF/water (4:1) was added and the reaction was run for 18 h at 60 °C. DCM was added to the reaction mixture and the degraded polymer was washed with $NaHCO_3$ (10 mL × 1) and purified via precipitation in cold methanol (15 mL × 2).

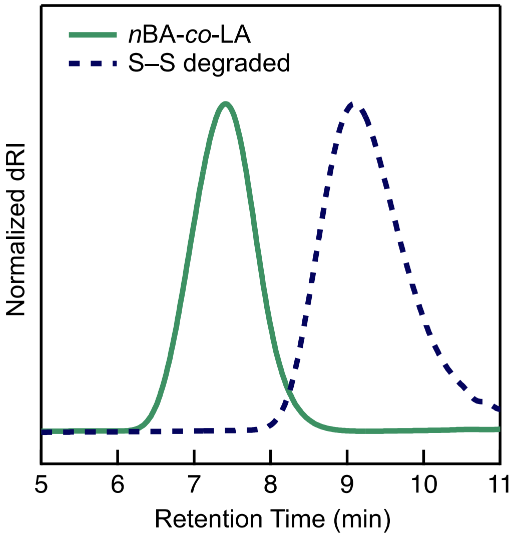


**Figure S11.** Representative SEC trace with normalized differential refractive index (dRI) detection for *n*BA-*co*-LA before (solid line) and after degradation (dashed line) with TCEP.

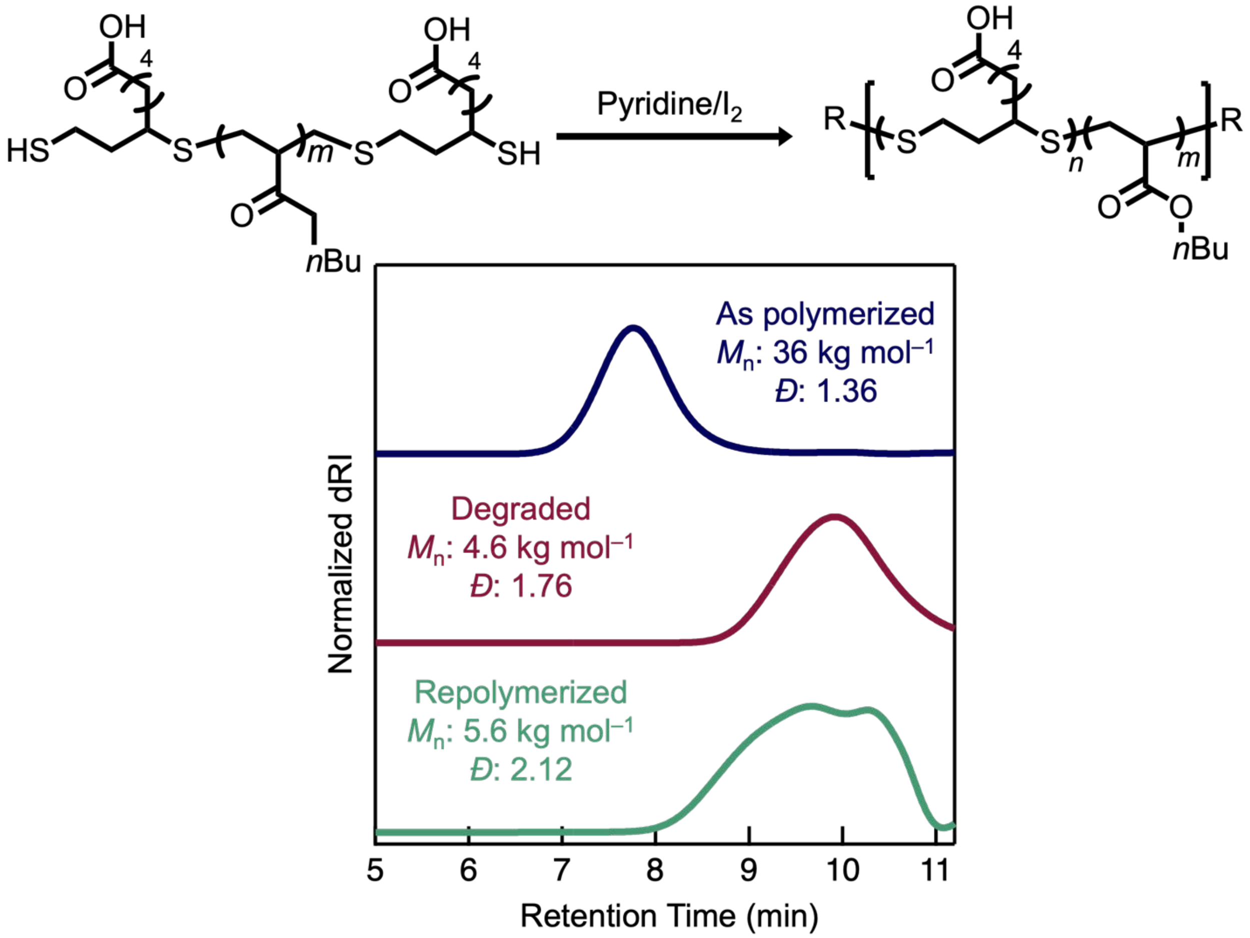


**Figure S12.** Representative SEC trace with normalized differential refractive index (dRI) detection for *n*BA-*co*-LA as synthesized (top), after degradation with TCEP (middle), and repolymerized with $I_2$/pyridine (bottom). Previously reported studies have shown the degraded species can be repolymerized through oxidation of the reactive thiol chain-ends.[2,3] The disulfide bonds should form in a step-growth fashion to recover the high molar mass polymers. However, the high density of carboxylic acid moieties in the system yielded an incomplete oxidation of the thiols due to the competing mechanism with the pyridine.

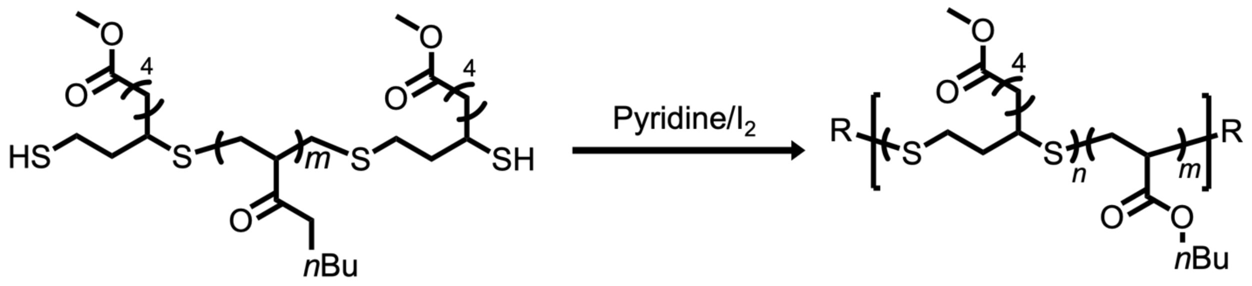


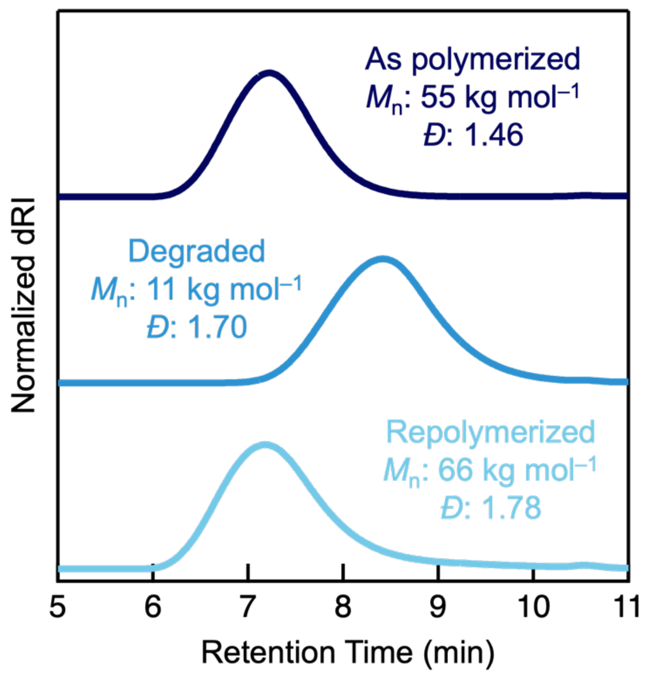


**Figure S13.** Representative SEC trace with normalized differential refractive index (dRI) detection for poly(butyl acrylate-*co*-methyl lipoate) (*n*BA-*co*-MLp) as synthesized (top) and after degradation with TCEP (middle), and repolymerized with $I_2$/pyridine (bottom). By using methyl lipoate for the repolymerization study, the oxidation reaction was able to go to quantitative completion.

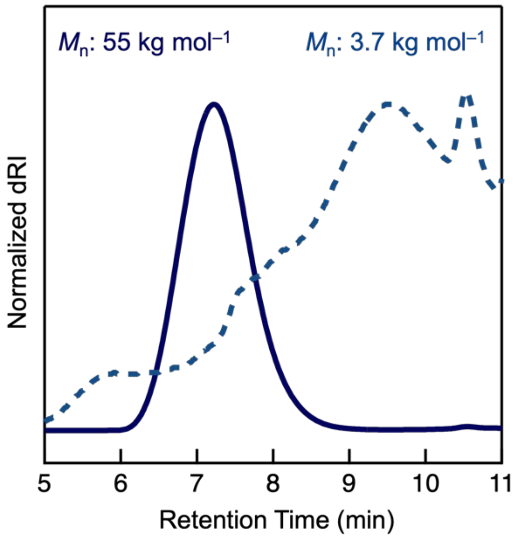


**Figure S14.** Representative SEC trace with normalized differential refractive index (dRI) detection for *n*BA-*co*-LA before (solid line) and after degradation (dashed line) with $NaBH_4$.

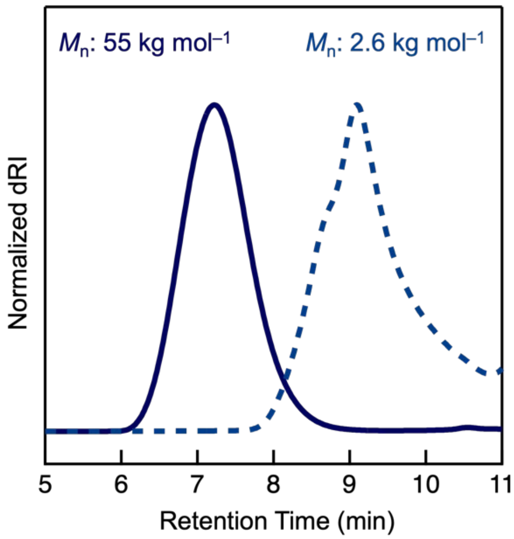


**Figure S15.** Representative SEC trace with normalized differential refractive index (dRI) detection for *n*BA-*co*-LA before (solid line) and after degradation (dashed line) with $AgNO_3$.

*Methylation of nBA-co-LA*

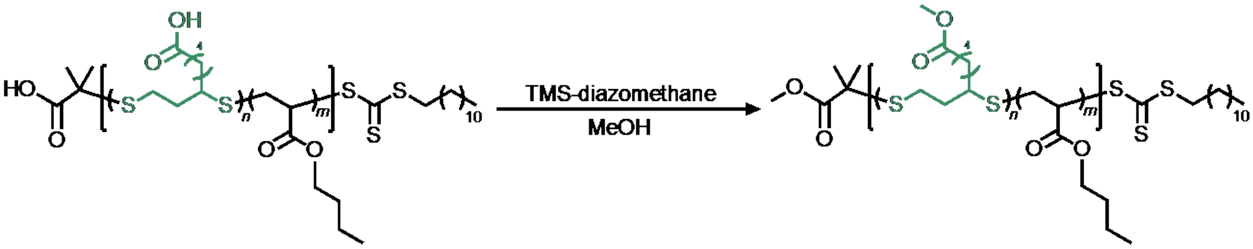


In a 4 mL dram vial, *n*BA-*co*-LA (100 mg, 0.001mmol) was dissolved in 2 mL of THF. To the stirring solution, methanol (0.2 mL, 4.9 mmol) was added followed by a dropwise addition of TMS-diazomethane (~5 drops, 0.2 M in ether) or until a light-yellow color remained in the stirring solution. The reaction mixture was left overnight to stir at room temperature and quenched with AcOH (6 drops) or until a colorless solution remained. The polymer was precipitated into cold methanol (50 mL × 5).

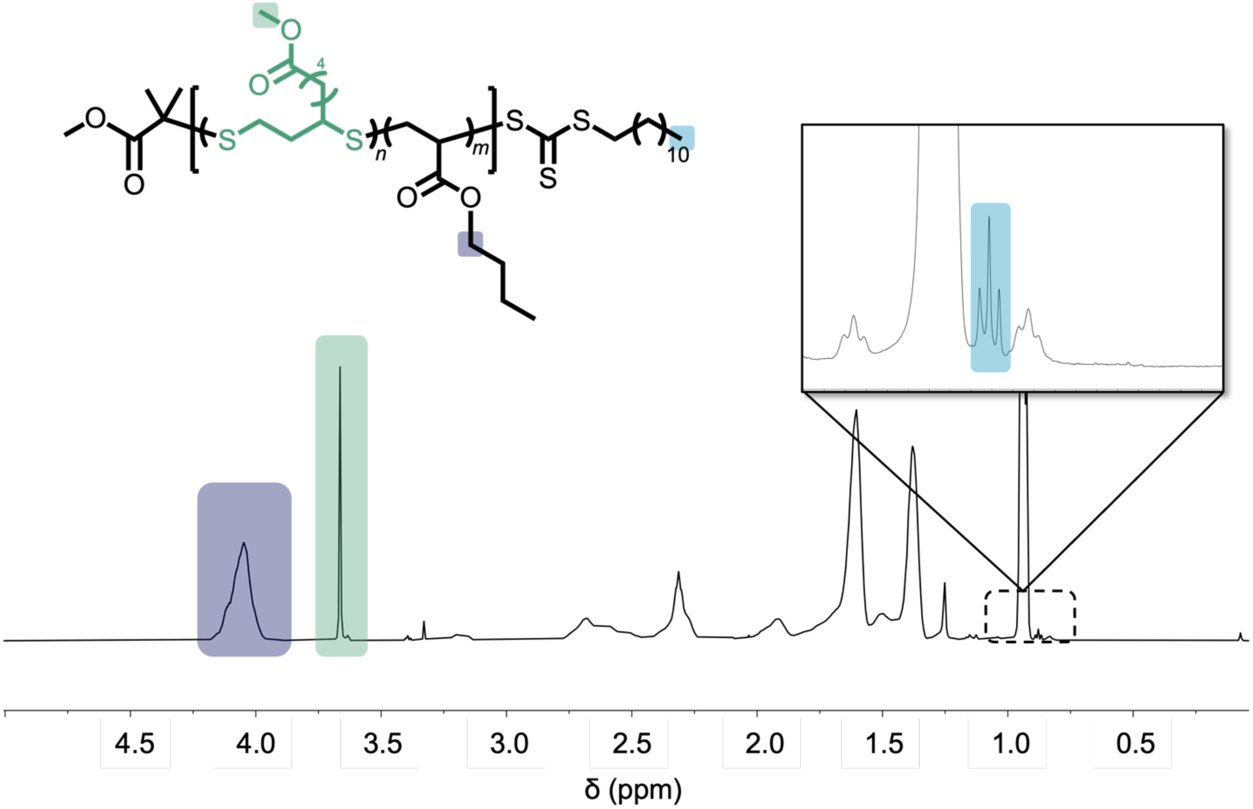


**Figure S16.** $^1$H NMR analysis of *n*BA-*co*-LA with characteristic resonances highlighted.

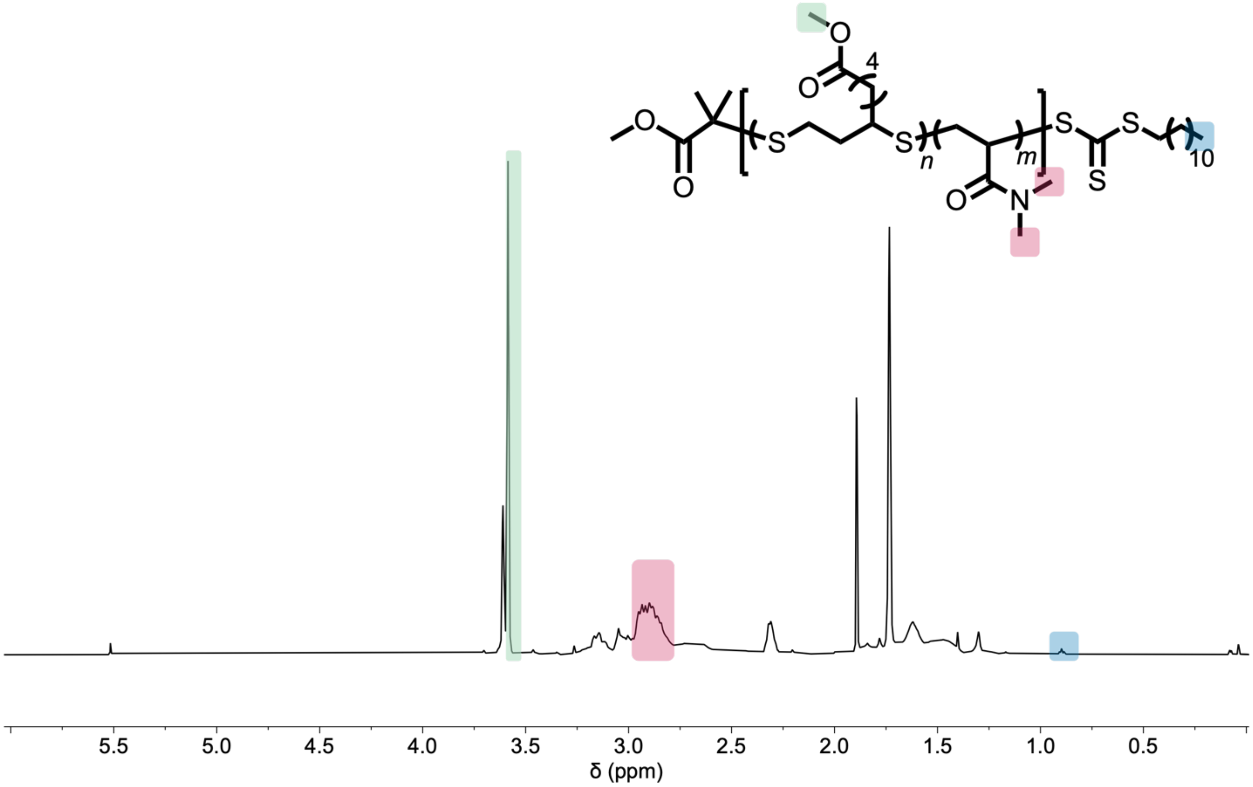


**Figure S17.** $^{1}$H NMR analysis of poly(dimethyl acrylamide-*co*-lipoic acid) after methylation with characteristic resonances highlighted.

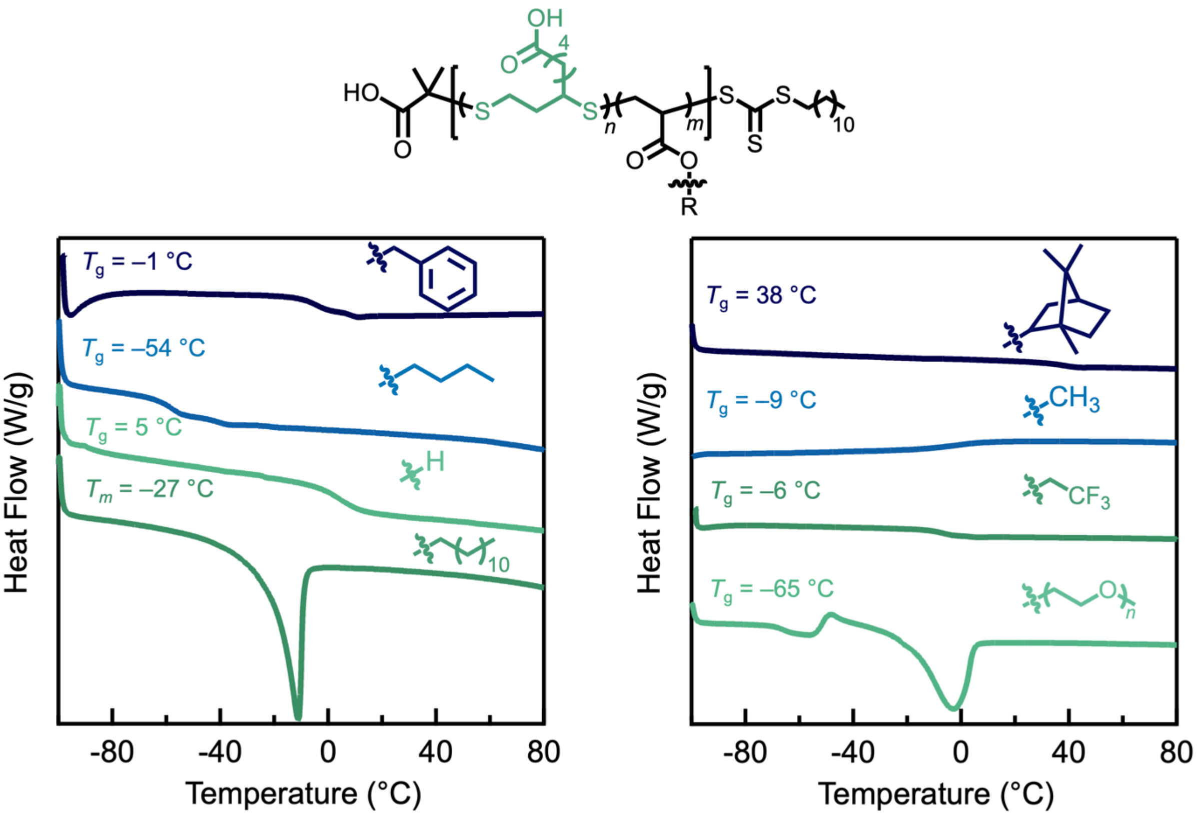


**Figure S18.** DSC thermograms of acrylate copolymers. The second heat, with a ramp rate of 10 °C $min^{-1}$, is plotted for clarity and plotted with exotherm up.

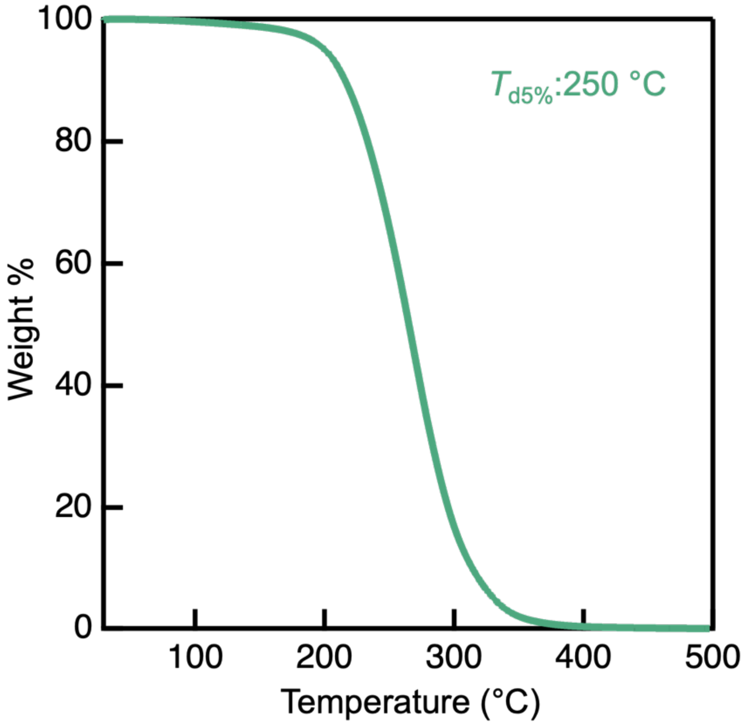


**Figure S19.** Representative TGA thermographs of *n*BA-*co*-LA copolymers in air with a ramp rate of 10 °C/ min .

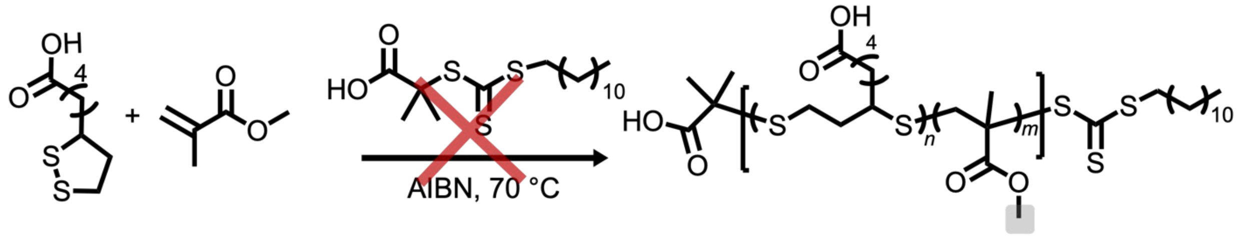


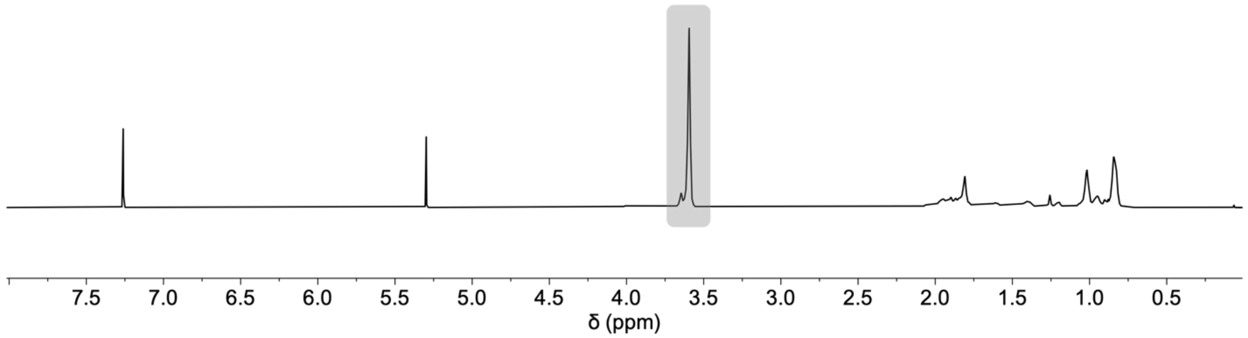


**Figure S20.** Copolymerization with lipoic acid and methyl methacrylate results in poly(methyl methacrylate) homopolymer due to incompatible reactivity with lipoic acid. $^1$H NMR analysis of characteristic resonances highlighted.

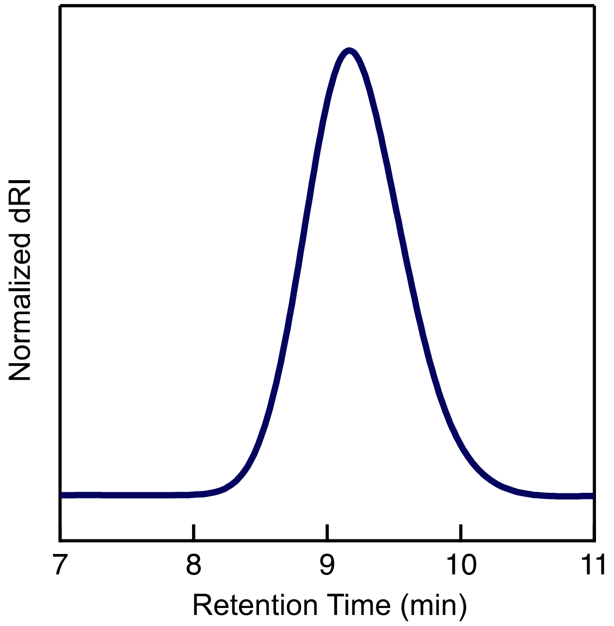


**Figure S21.** SEC trace with normalized differential refractive index (dRI) detection for reaction depicted in Figure S20.

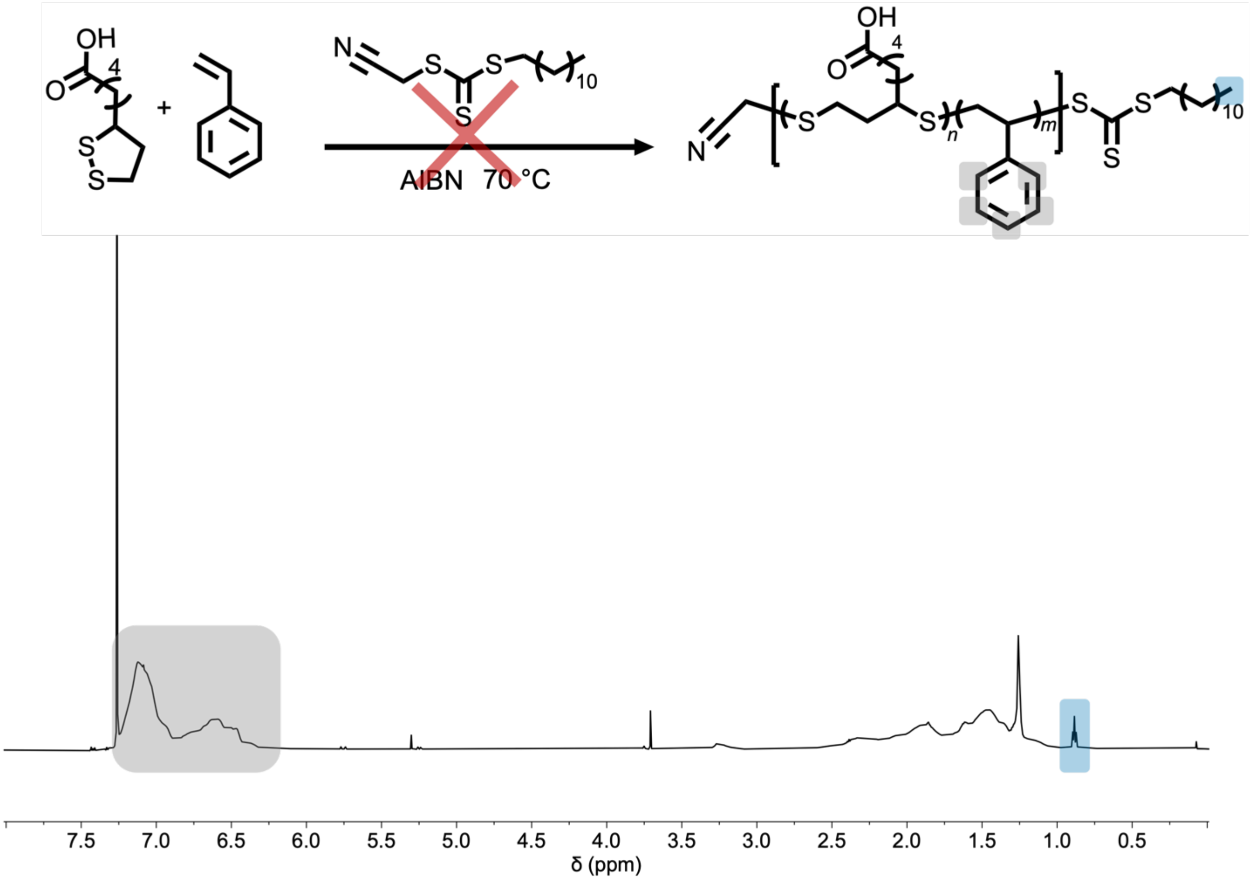


**Figure S22.** Copolymerization with lipoic acid and styrene results in polystyrene homopolymer due to incompatible reactivity with lipoic acid. $^{1}$H NMR analysis of characteristic resonances highlighted.

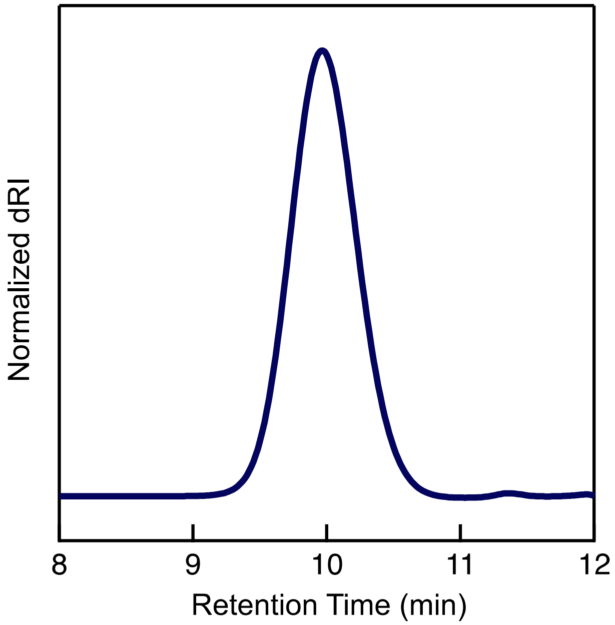


**Figure S23.** SEC trace with normalized differential refractive index (dRI) detection for reaction depicted in Figure S22.

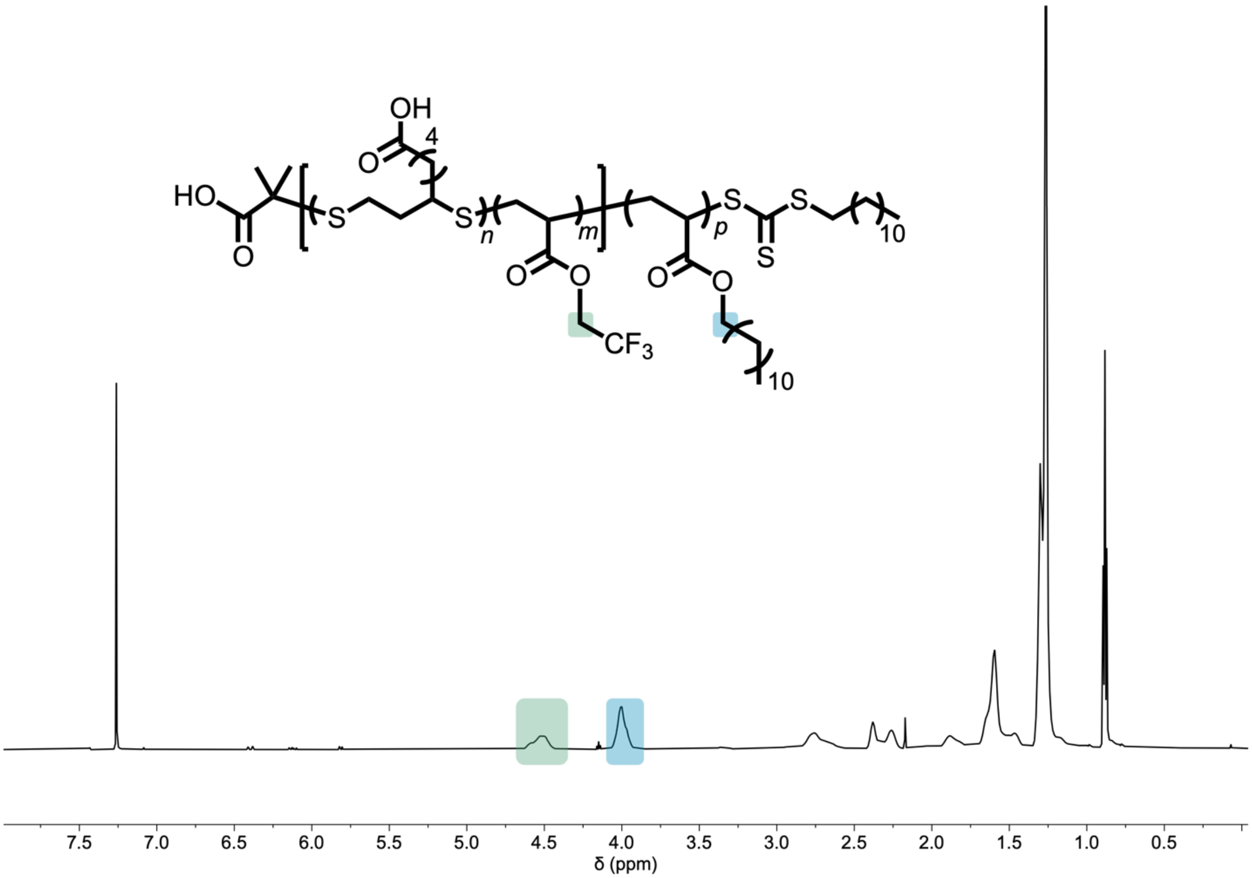


**Figure S24.** $^1$H NMR analysis of [LA-*co*-TFA]-*b*-DA diblock with characteristic resonances highlighted.

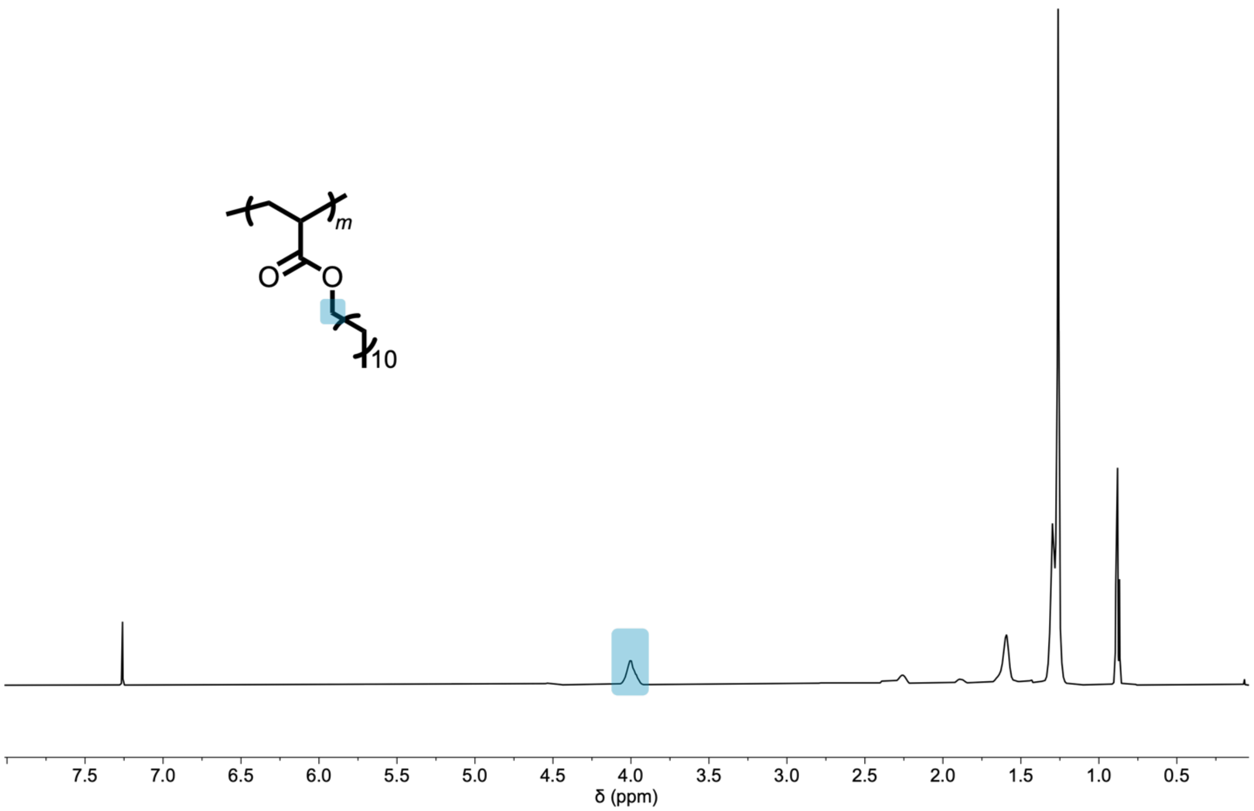

**Figure S25.** $^{1}$H NMR analysis of DA oligomer after fractional precipitation with characteristic resonances highlighted.

*Synthesis of PEG-CTA*

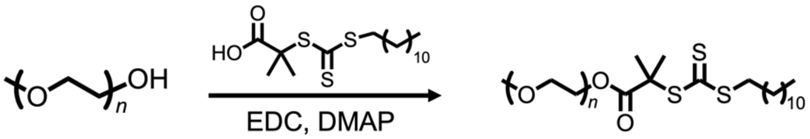


In a 100 mL round bottom flask, DTT ( 0.675 g, 1.8 mmol), PEG (4.445 g, 2.2 mmol), and DMAP (0.272 g, 2.2 mmol) were dissolved in 50 mL of DCM. The reaction flask was cooled to 0 °C in an ice bath and a solution of EDC (0.345 g, 2.2 mmol) was slowly added to the stirring solution. The reaction flask was brought to room temperature and left to stir for 16 h. The resulting solution was condensed and purified via column chromatography (100% hexanes to 100% ethyl acetate) to yield a bright yellow solid. $M_{n,SEC}$: 1.8 kg mol $^{-1}$, $Đ$ =1.04

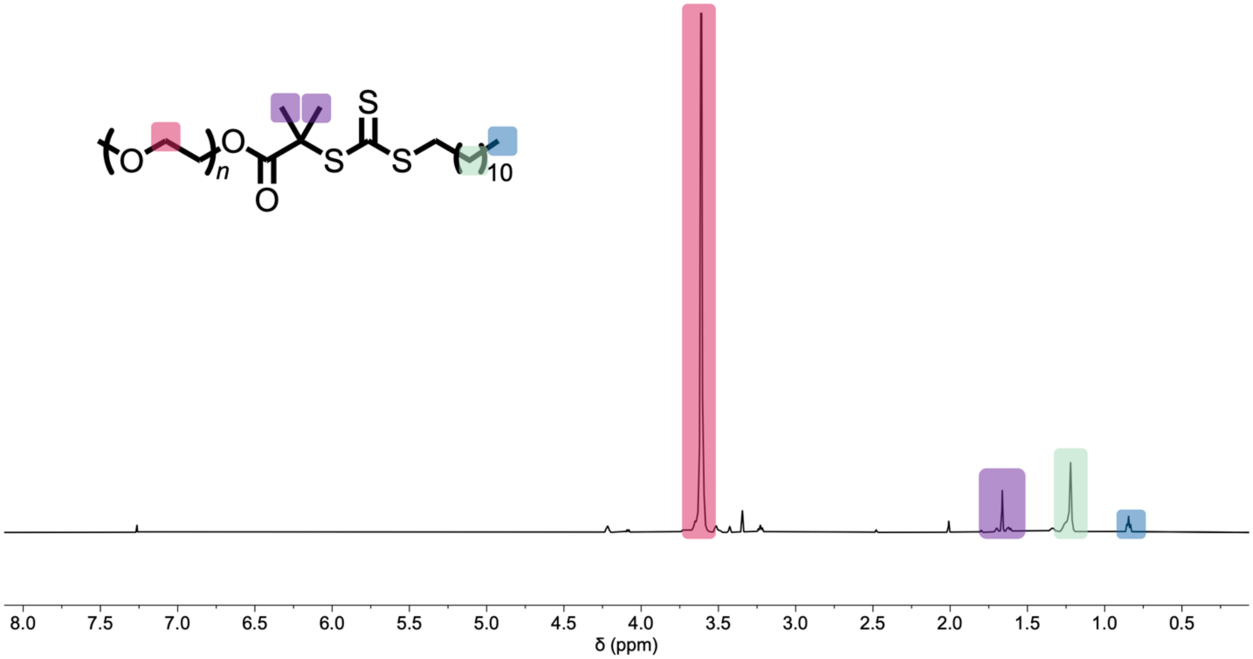


**Figure S26.** $^{1}$H NMR analysis of PEG-CTA with characteristic resonances highlighted.

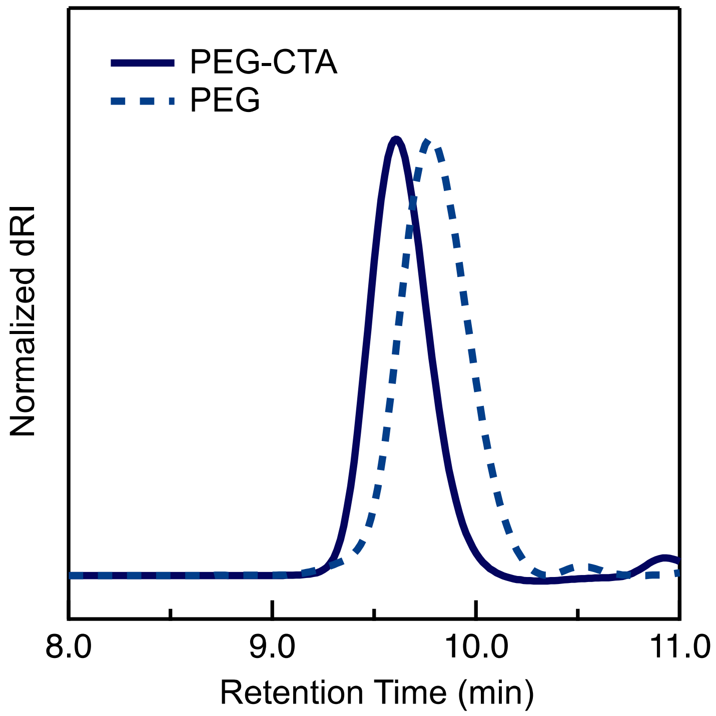


**Figure S27.** SEC trace with normalized differential refractive index (dRI) detection for PEG-CTA.

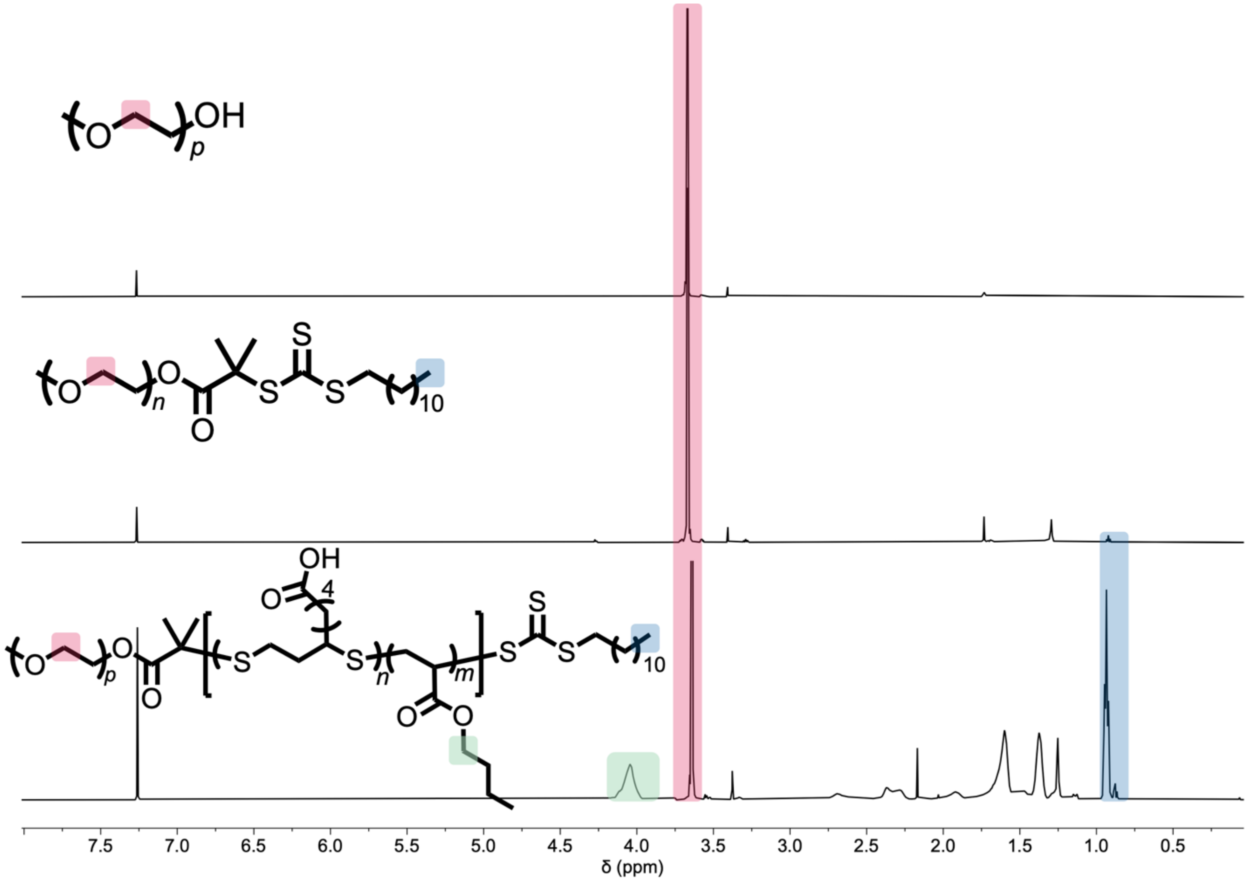


**Figure S28.** $^{1}$H NMR analysis of PEG (top), PEG-CTA (middle) and PEG-*b*-(LA-*co*-*n*BA) diblock (bottom) with characteristic resonances highlighted.

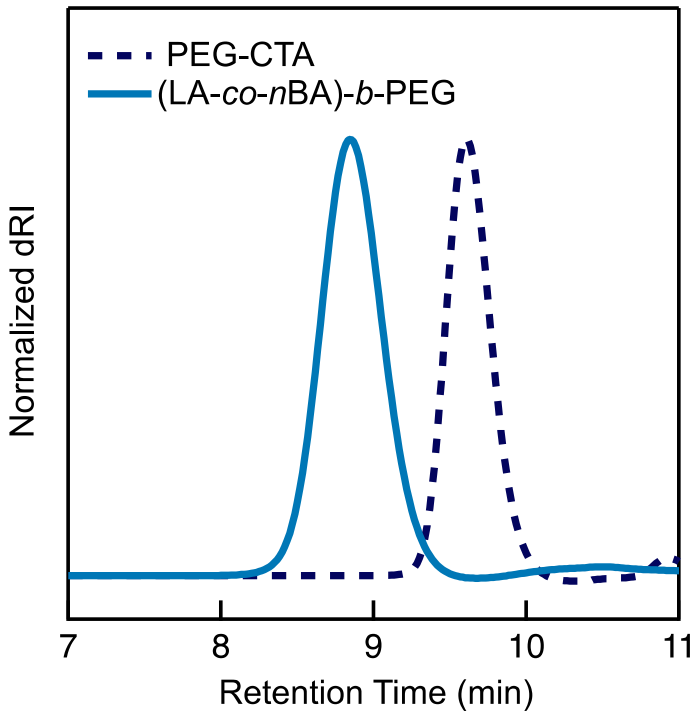


**Figure S29.** SEC trace with normalized differential refractive index (dRI) detection for PEG-CTA and PEG-*b*-(LA-*co*-*n*BA) ($M_{n,SEC}$: 8.4 kg mol $^{-1}$, $Đ$ =1.08).

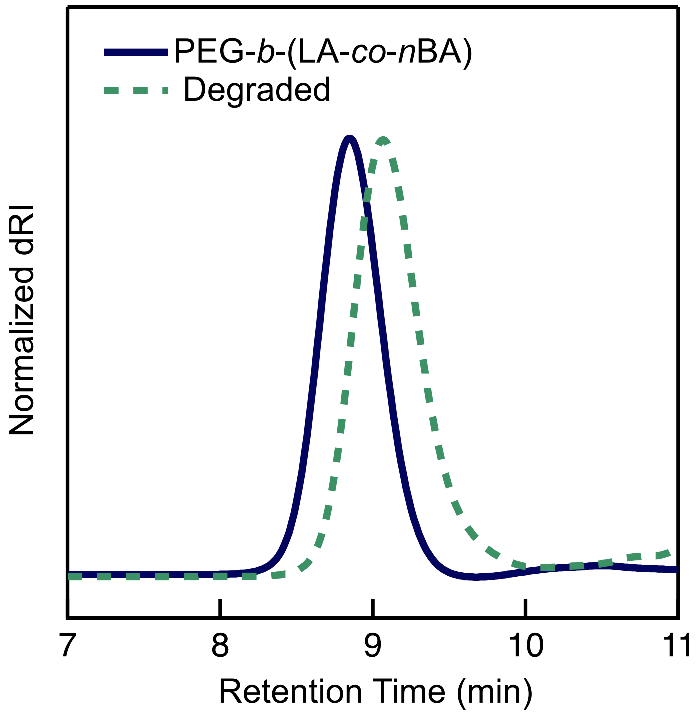


**Figure S30.** SEC trace with normalized differential refractive index (dRI) detection for PEG-*b*-(LA-*co*-*n*BA) before and after degradation. ($M_{n,SEC}$: 5.8 kg mol$^{-1}$, $Đ$ =1.20).

*Micelle Formation and Dynamic Light Scattering (DLS)*

A stock solution of polymer (~2.2 mg) was dissolved in 100 µL of THF. The micelles were prepared by rapidly mixing the THF solution with 3 mL of Milli-Q water.

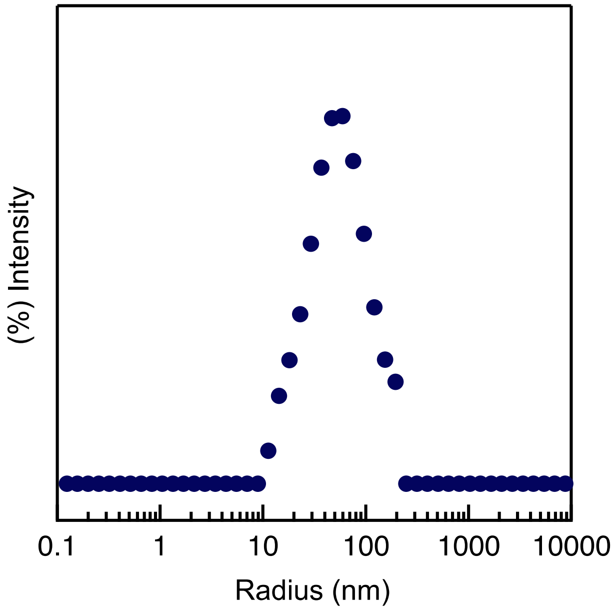

**Figure S31.** DLS trace for PEG-*b*-(LA-*co*-*n*BA) in aqueous medium.

## REFERENCES


(1) Bell, C. A.; Hedir, G. G.; O'Reilly, R. K.; Dove, A. P. Controlling the Synthesis of Degradable Vinyl Polymers by Xanthate-Mediated Polymerization. *Polym. Chem.* **2015**, *6* (42), 7447–7454. https://doi.org/10.1039/C5PY01156F.

(2) Kiel, G. R.; Lundberg, D. J.; Prince, E.; Husted, K. E. L.; Johnson, A. M.; Lensch, V.; Li, S.; Shieh, P.; Johnson, J. A. Cleavable Comonomers for Chemically Recyclable Polystyrene: A General Approach to Vinyl Polymer Circularity. *J. Am. Chem. Soc.* **2022**, *144* (28), 12979–12988. https://doi.org/10.1021/jacs.2c05374.

(3) Albanese, K. R.; Okayama, Y.; Morris, P. T.; Gerst, M.; Gupta, R.; Speros, J. C.; Hawker, C. J.; Choi, C.; De Alaniz, J. R.; Bates, C. M. Building Tunable Degradation into High-Performance Poly(Acrylate) Pressure-Sensitive Adhesives. *ACS Macro Lett.* **2023**, *12* (6), 787–793. https://doi.org/10.1021/acsmacrolett.3c00204.
